# U.S. winter wheat yield loss attributed to compound hot-dry-windy events

**DOI:** 10.1038/s41467-022-34947-6

**Published:** 2022-11-24

**Authors:** Haidong Zhao, Lina Zhang, M. B. Kirkham, Stephen M. Welch, John W. Nielsen-Gammon, Guihua Bai, Jiebo Luo, Daniel A. Andresen, Charles W. Rice, Nenghan Wan, Romulo P. Lollato, Dianfeng Zheng, Prasanna H. Gowda, Xiaomao Lin

**Affiliations:** 1 Department of Agronomy, Kansas State University, 2004 Throckmorton Hall, Plant Sciences Center, Manhattan, KS 66506 USA; 2 Kansas Climate Center, Kansas State University, 2108 Throckmorton Hall, Plant Sciences Center, Manhattan, KS 66506 USA; 3 Department of Atmospheric Sciences, Texas A&M University, College Station, TX 77843 USA; 4 Hard Winter Wheat Genetics Research Unit, USDA–ARS, Kansas State University, Manhattan, KS 66506 USA; 5 Department of Computer Science, University of Rochester, Rochester, NY 14627 USA; 6 Department of Computer Science, Kansas State University, Manhattan, KS 66506 USA; 7 College of Coastal Agriculture Sciences, Guangdong Ocean University, Zhanjiang, Guangdong, 524088 China; 8 USDA, Agricultural Research Service, Southeast Area, Stoneville, MS 38776 USA

**Keywords:** Climate-change impacts, Environmental impact

## Abstract

Climate extremes cause significant winter wheat yield loss and can cause much greater impacts than single extremes in isolation when multiple extremes occur simultaneously. Here we show that compound hot-dry-windy events (HDW) significantly increased in the U.S. Great Plains from 1982 to 2020. These HDW events were the most impactful drivers for wheat yield loss, accounting for a 4% yield reduction per 10 h of HDW during heading to maturity. Current HDW trends are associated with yield reduction rates of up to 0.09 t ha^−1^ per decade and HDW variations are atmospheric-bridged with the Pacific Decadal Oscillation. We quantify the “yield shock”, which is spatially distributed, with the losses in severely HDW-affected areas, presumably the same areas affected by the Dust Bowl of the 1930s. Our findings indicate that compound HDW, which traditional risk assessments overlooked, have significant implications for the U.S. winter wheat production and beyond.

## Abstract (web summary)

The authors show that in recent decades compound climate extremes (i.e., hot, dry, and windy events) have increased and have reduced winter wheat yields in the U.S. Great Plains. The area most affected is the same area as that in 1930s Dust Bowl.

## Introduction

Despite continued increase worldwide, wheat yields in some parts of the world have stagnated and even collapsed^1,2^, raising serious concerns associated with sustainable production in the face of climate change^3,4^. Recent studies have examined the risks of single, extreme climatic events in causing yield losses^5^, such as drought (precipitation-based)^6,7^, heat waves^8^, or cold events^9^. There are also studies that have examined the effects of multiple extreme climate events on agricultural yields in conjunction, but not as compound events^10,11^. However, events that combine adverse climate variables, not all of which are necessarily extremes, could lead to more significant impacts on wheat yields than any single type of shock acting alone. The Intergovernmental Panel on Climate Change (IPCC) Special Report on Climate Extremes (SREX) in 2012^3^ first defined compound events as “(1) two or more extreme events occurring simultaneously (spatial) or successively (temporal), (2) combinations of extreme events with underlying conditions that amplify the impact of the events, or (3) combination of events that are not themselves extremes but lead to an extreme event or impact when combined.” This definition has recently evolved into “a combination of multiple drivers and/or hazards that contribute to societal or environmental risk”^12–14^. With respect to such compound events, few efforts have evaluated their impacts, and most of the existing research has focused on changes or distribution shifts of crop water supply and temperature. The simultaneous combination of high temperature, low relative humidity, and high wind events in the near-surface atmospheric boundary layer [hereafter referred to as hot-dry-windy events (HDW)] poses a particular climate risk to global crops^11,15,16^, especially in the United States Great Plains that experience irregular, hot, dry, and windy conditions coupled together^17–19^. For example, a decade of drought occurred in the 1930s in the U.S. and this decadal drought desiccated much of the agricultural land surface of the U.S. Great Plains causing one of the most severe environmental and social catastrophes in the world, namely the 1930s “Dust Bowl”^20^. The Dust Bowl area is the world’s largest, contiguous land mass with low rainfall^9^ and is the U.S. heartland for winter wheat production. In this agricultural area, the spring-summer HDW events have statistically increased since the 1950s^17,21^. However, to our knowledge, the consequence of HDW with respect to hard winter wheat production in the U.S. has not yet been assessed.

Previously, the standard practice in determining climate-crop relationships for winter wheat has been to partition its growth stages by fall, winter, and spring calendar seasons^9,19^. The use of such fixed calendars with subsets of seasons or months, however, might potentially misrepresent the climate-crop association^22^ due to varied local phenological timings, stage-specific crop sensitivity to adverse weather, differences in crop management, and spatially variable regional climates^7^. Hence, unlike most studies using annual and/or seasonal climate extreme indices, we used climate indices directly derived from the winter wheat life cycle, which we divided into three phenological stages: planting to jointing (PT-JT), jointing to heading (JT-HD), and heading to maturity (HD-MT) based on phenological data (Supplementary Text 1; Supplementary Figs. 1 and 2; and Supplementary Table 1). We digitized most of these phenological data from hard copies of Winter Wheat Performance Tests Reports or scanned imageries across South Dakota (SD), Nebraska (NE), Colorado (CO), Kansas (KS), Oklahoma (OK), and Texas (TX) in the U.S. Great Plains. The subdivision of the growing season based on crop phenology has a biological meaning as PT-JT represents wheat conditions associated with crop establishment, tillering, and winter survival; JT-HD encompasses a large portion of the critical period for grain number determination; and HD-MT corresponds to both the later portion of grain number determination and the majority of the individual grain weight determination^23^.

Crop growth and development as well as yield formulation showed a threshold response to climate variation^7^ including temperature^5,24,25^, atmospheric humidity^26^, and ambient wind speed^27^. We defined hourly HDW events as the hours of co-occurrence of temperature (T) ≥ 32 °C, relative humidity (RH) ≤ 30%, and wind speed (U) ≥ 7 m s^−1^ at 10 m above the ground, as described in previous studies^21,28^. Crop water stress can be due to excessive loss of water or an inadequate soil water supply^29^. For the former, the main cause is a rapid transpiration of the crop, often occurring in less than a few hours due to atmospheric dryness. Specifically, low atmospheric humidity creates a demand that pulls water from plants and causes excessive transpiration which induces a water deficit in the crop that restricts growth. The latter, however, can result from long-term precipitation deficits or inadequate soil moisture over a period of several days^30^. In recent studies^31,32^, the main focus was on the relationship between crop yields and inadequate soil water supply determined by precipitation or soil moisture. However, some studies have indicated that atmospheric dryness, a rapid water demand occurring in less than a few hours, impacts severely plants even if soil water supply is adequate^33^ and concluded that low relative humidity in the atmosphere can be more important to gauge the ecosystem impacts of dryness^34,35^. In our study, the hot-dry-windy (HDW) combination is a transient and compound extreme event including atmospheric dryness that commonly occurs within several hours. Hence, we used low relative humidity to define a dry event.

We focused primarily on the effects of often overlooked hourly HDW events during HD-MT (HDW_HD-MT_), i.e., grain-filling periods on 1982–2020 winter wheat yields in the U.S. winter wheat belt that includes the states of SD, NE, CO, KS, OK, and TX. These states produce nearly all the high-quality hard red winter wheat in the U.S.^9^.

## Results and discussion

### HDW trends

**Fig. 1:**
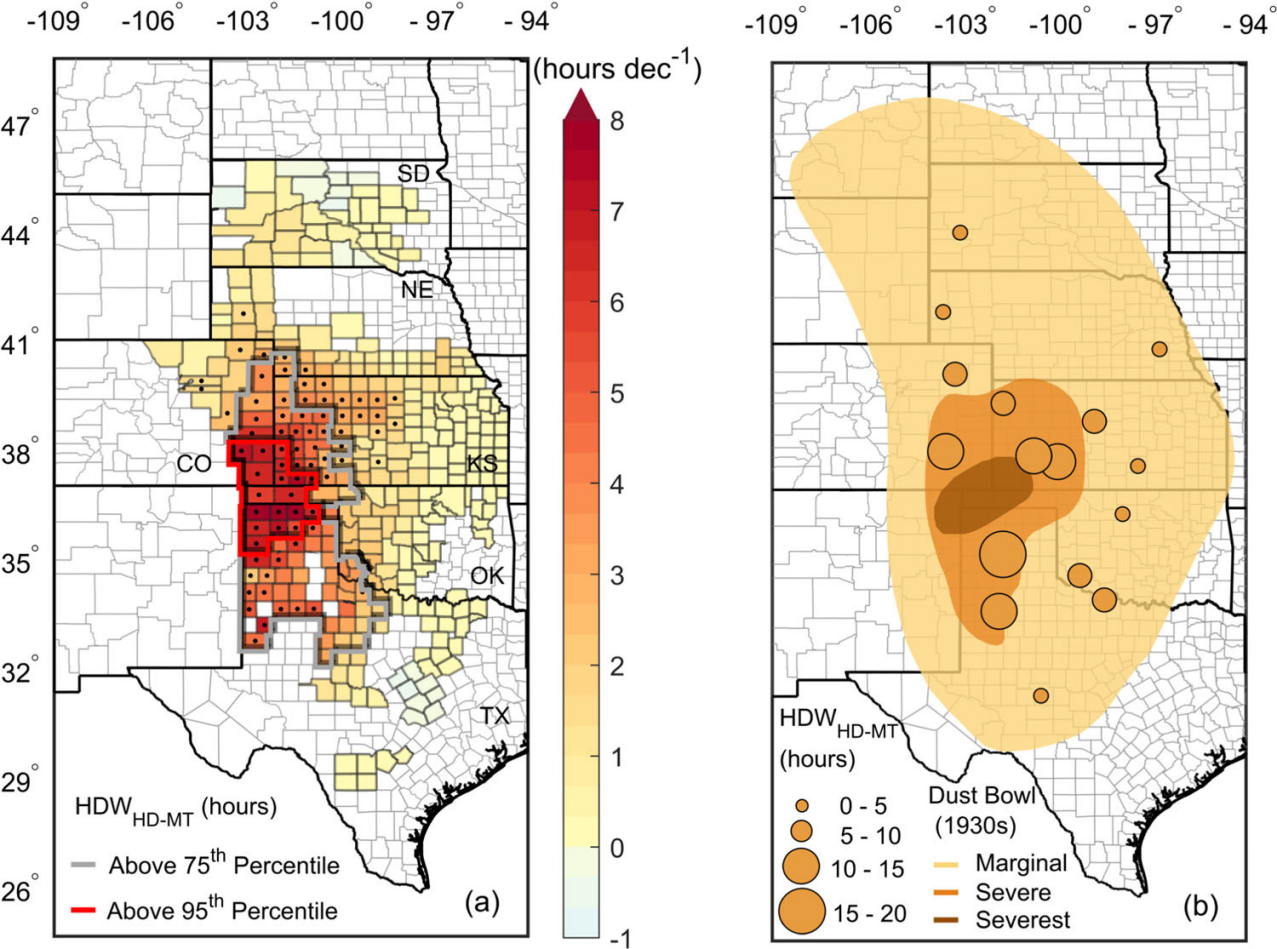
Hot-dry-windy events (HDW) trends (hours per decade) during the heading-maturity stage (HDW_HD-MT_) from 1982–2020 and annual average of HDW_HD-MT_ from climate observing stations. **a** Annual HDW_HD-MT_ trends for all winter wheat counties; the black dots indicate that HDW trends were statistically significant; and the gray and red boundaries indicate HDW areas above the 75th (>5 h) and 95th (>15 h) percentiles of annual averaged HDW_HD-MT_ hours in our study domain including South Dakota (SD), Nebraska (NE), Colorado (CO), Kansas (KS), Oklahoma (OK), and Texas (TX). **b** Annual averaged HDW_HD-MT_ estimated from climate observing stations; the shading indicates marginal, severe, and severest 1930s Dust Bowl areas.

We found that the main, annual HDW-affected areas occurred in southwest Kansas and the panhandle areas of Oklahoma and Texas in the U.S. (Fig. 1a red and gray boundaries). These frequent HDW-affected areas were also verified by independent climate observing stations (Fig. 1b) (Supplementary Text 2 and 3). More importantly, the majority of the upward HDW_HD-MT_ trends at county levels were statistically significant and were located inside the area above the 75th percentile of annual HDW_HD-MT_ (Fig. 1a). Both the frequent, annual HDW-affected area and the locations with the greatest upward HDW_HD-MT_ trend from 1982 to 2020 were the same as the Dust Bowl-affected locales of the 1930s^20,36^ (Fig. 1b). Such averaged spatial patterns and increasing trend patterns of HDW were consistent with limited, individual station observations from 1982 to 2020 (Supplementary Text 3 and Supplementary Figs. 3 and 4).

Upward trends in all relevant individual extreme climate events (Supplementary Fig. 5) increased as did the frequency of occurrence of HDW_HD-MT_ for most of the counties across all six states (Fig. 1a) (Supplementary Fig. 6). Increased trends of HDW_HD-MT_ were statistically significant in the most HDW-affected areas at a rate up to 8 h per decade (Fig. 1a). A partial correlation analysis conducted to examine the most critical factor modulating the occurrence of HDW, suggests that high temperature events are a major control variable (Supplementary Fig. 7).

### HDW impacts on wheat yields

**Fig. 2:**
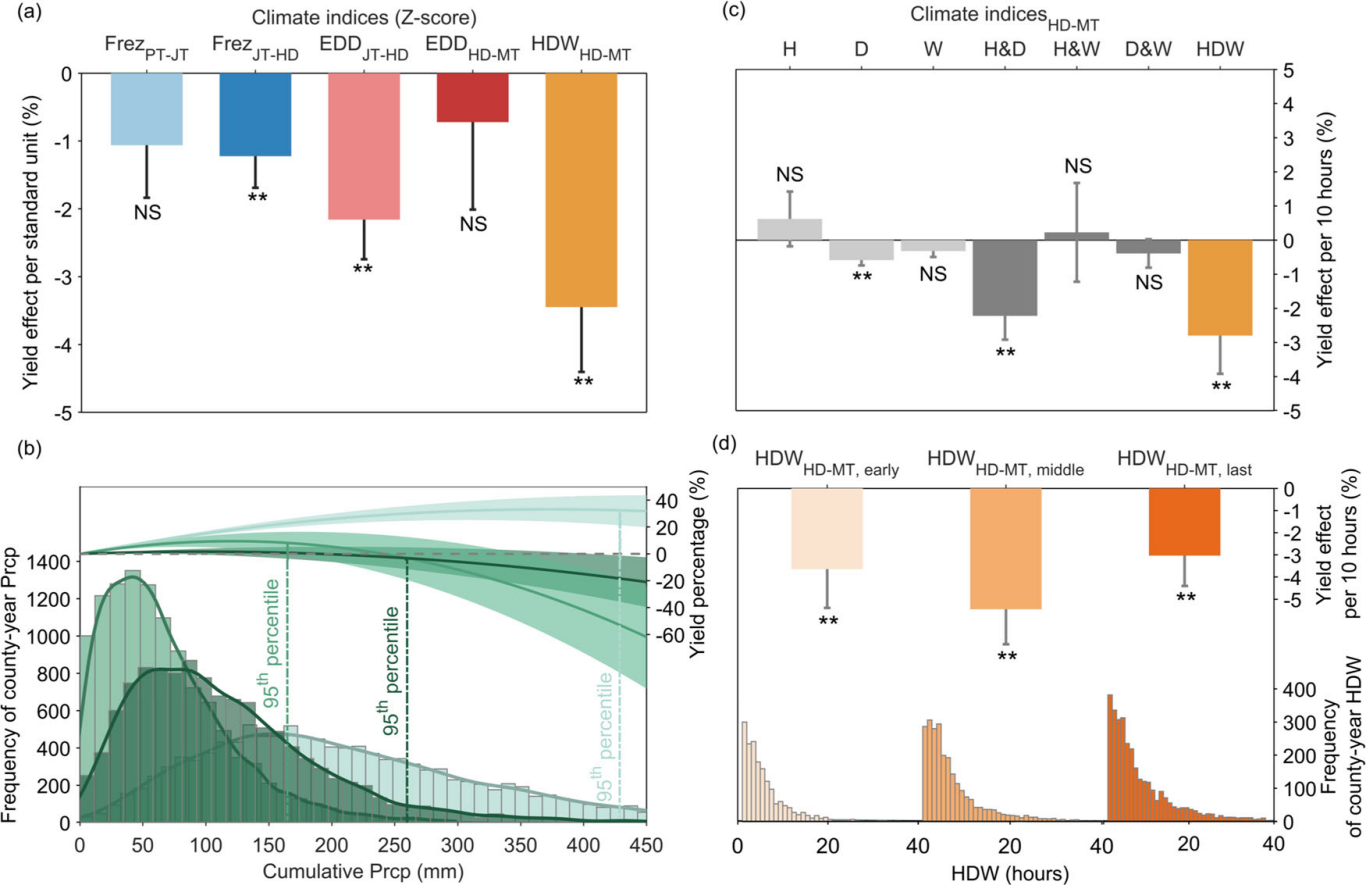
Estimated effects of climate indices on winter wheat yields relative to the average yield. **a** The yield effects are expressed as a percent of area-weighted average yield (grand average = 2.4 t ha^−1^) across our study domain per standard unit of climate indices (Supplementary Table 2). One standard unit of climate indices in their distributions across the study domain is 14.77 days for Frez_PT-JT_, 0.89 days for Frez_JT-HD_, 0.35 °C days for EDD_JT-HD_, 2.81 °C days for EDD_HD-MT_, and 9.03 h for HDW. Frez stands for freezing; EDD stands for extreme degree days; HDW stands for hot-dry-windy events. PT-JT is the planting to jointing stage; JT-HD is the jointing to heading stage; HD-MT is the heading to maturity stage. **b** The percent change in yield as a function of the cumulative precipitation (Prcp) by growth stages. The shaded bands represent the one adjusted standard error of regression in the original units (mm). Histograms show the frequency of county-year accumulated precipitation for specific phenological stages. The three vertical lines represent the 95th percentile of Prcp for corresponding phenological stages. **c** Yield effects of climate indices including individual extreme events (hot, H; dry, D; wind, W), bivariate events (hot-dry, H&D; hot-windy, H&W; dry-windy, D&W), and trivariate compound events (hot-dry-windy, HDW). **d** Yield sensitivity to HDW (bars) and frequency of county-year HDW (histograms) during early, middle, and last sub-stages from HD to MT stage. The error bars indicate one clustering standard error (SE) and asterisks denote the significance (**p ≤ 0.05); NS, not significant (p > 0.05).

To assess impacts of HDW and single-variable climate extremes on wheat yield variability at different phenological stages, a linear mixed-effects model^37^ was constructed for climate-crop interaction mechanics including freezing days (Frez, days), extreme degree days (EDD, °C days), precipitation (Prcp, mm), and HDW (hours) (Supplementary Fig. 1) in the U.S. winter wheat belt^7,38^. In the following, standardized climate indices^39^ are referred to as ‘standard units’ to explain their effects on yields (Fig. 2a and Supplementary Table 2). The linear mixed-effects model was also tested with untransformed climate indices in their original units (Supplementary Table 3) to directly interpret impacts using their natural scales (see Methods for model equations and details). The results showed that 59% of the variation in yields can be explained by climate indices (*r*^2^ = 0.59). HDW_HD-MT_ significantly dominated yield variability with a 3.5% yield loss per standard unit or a 4% yield loss per 10 h (*p*_adj_ < 0.001), followed by EDD_JT-HD_ (2.2% yield loss per standard unit) (*p*_adj_ < 0.001) (Fig. 2a and Supplementary Tables 2 and 3). Next, we compared impacts of individual, bivariate, and trivariate compound events on yields. We found that hot (H) and compound hot-winds (H&W) did not significantly impact wheat yields, nor did the windy (W) and compound (D&W) events but yields were significantly reduced when the compound hot-dry (H&D) event occurs (Fig. 2c). Interaction of high temperature and dry events reduces the storage capacity of the wheat grain by decreasing the number of cells, starch granules, the duration of cell division, and dry matter accumulation^40^. Importantly, we found that HDW events show the largest negative influence on wheat yields (Fig. 2c). Dry winds accompanying high temperature damage plant parts that actively and photosynthetically supply the head (e.g., the flag leaf in wheat), resulting in sterile florets and yield loss^41,42^. The impacts of the HDW on wheat yields were further assessed during early, middle, and last sub-stages of the HD-MT stage. We found that the HDW occurrence during the middle sub-stage (around the floral stage) of the HD-MT stage shows the largest influence on yields (Fig. 2d).

**Fig. 3:**
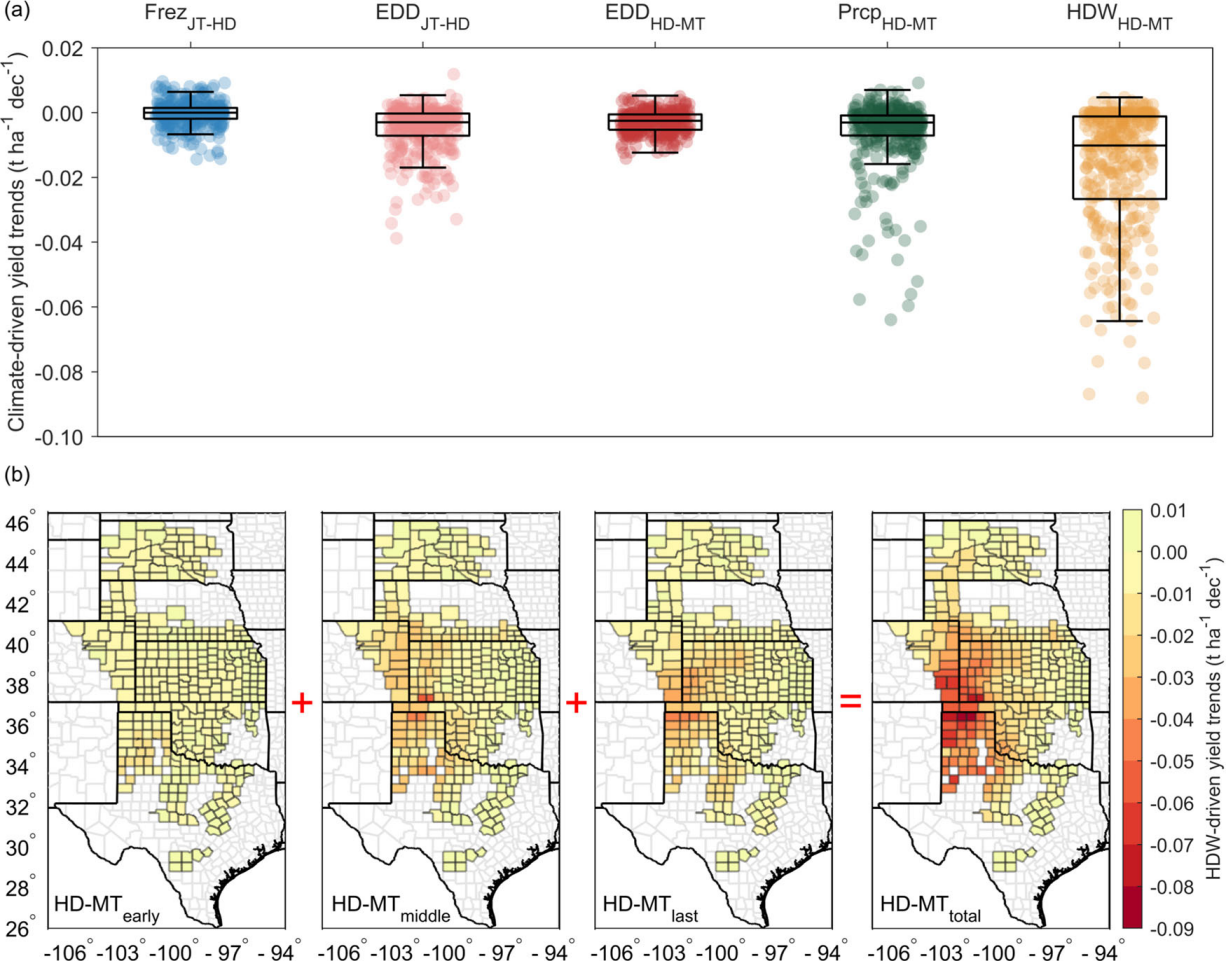
Climate contributions to yield trends from 1982 to 2020. **a** Yield trends associated with freezing days (Frez) and extreme degree days (EDD) during the jointing (JT) to heading (HD) stages, and with the EDD, precipitation (Prcp), and hot-dry-windy (HDW) during the heading (HD) to maturity (MT) stages. Box plots indicate median (middle line), 25th, 75th percentile (box) and 5th and 95th percentile (whiskers). Each dot represents individual county-level climate-driven yield trends. **b** The spatial distribution of HDW-driven yield trends during early, middle, and last sub-stages of HD-MT stage and total HD-MT stage.

The climate-driven yield trends (Fig. 3) indicated that single climate extreme events provided negative impacts during phenological stages. HDW_HD-MT_ are associated with a yield loss at a rate of up to 0.09 t ha^−1^ per decade in counties severely affected by HDW_HD-MT_ (Fig. 3a), suggesting HDW_HD-MT_ were the most influential drivers among those studied associated with yield variability (or loss) in the U.S. winter wheat belt. We further disentangled the total effects of HDW trends into three sub-stages during HD-MT stage and found the severe impacts mainly occur during the middle and last sub-stages (Fig. 3b). This was generally consistent with the results from heat shock experiments in a controlled environment^43^ where it is concluded that the middle and last sub-stages cause more damage and reduction compared to the early sub-stage of heading to maturity in wheat yield. These severe HDW_HD-MT_ impacts were spatially located in the same area as the Dust Bowl that occurred in the 1930s (Fig. 3b).

### HDW teleconnections relations

**Fig. 4:**
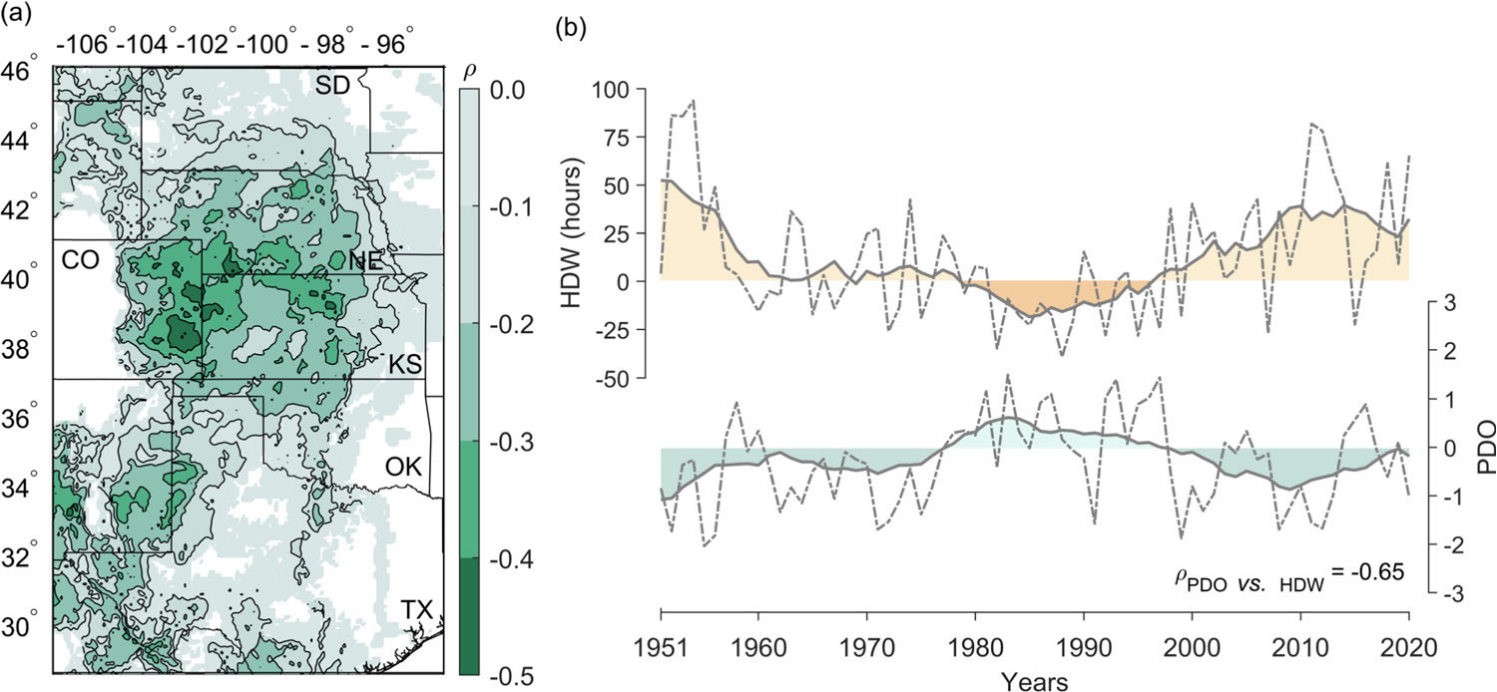
The partial Spearman’s rank correlation coefficient between Pacific Decadal Oscillation (PDO) and hot-dry-windy (HDW) anomalies (after detrended) during April-June from 1951 to 2020 in our study domains including South Dakota (SD), Nebraska (NE), Colorado (CO), Kansas (KS), Oklahoma (OK), and Texas (TX). **a** The spatial correlation coefficient between PDO and HDW anomalies where the temperature effect was controlled. **b** Time series of both PDO and HDW detrended anomalies. The HDW anomaly time series was averaged from the region with above the 95th percentile of annual averaged HDW (Fig. 1a). Dashed lines represent the raw anomaly (base period for anomaly: 1981–2010) and solid lines with shadowed areas represent the smoothed time series. The smoothing was conducted by a 9-year moving average. *ρ* refers to correlation coefficient.

We think the Pacific Decadal Oscillation (PDO)^44^, as displayed in Fig. 4a (color shading), is the atmospheric bridge and provides natural decadal changes in the climate system for these severe HDW_HD-MT_ counties, where they also have high rate of increase during the past 40 years (Fig. 1a, gray colors). A decadal variation of HDW_HD-MT_ signals showed a clear opposite pattern correlated with the PDO (*ρ* = −0.65) from 1951 to 2020 (Fig. 4b). The 9-year moving average trends (Fig. 4b) coupled with PDO variations^44,45^ suggested that the approximate HDW_HD-MT_ periodicity was >40 years and the HDW_HD-MT_ anomalies mostly fluctuated from −30 to 30 h during April to June. Such decadal variations of HDW_HD-MT_ provide important implications for U.S. wheat production (Figs. 2–4).

### Climate-driven yield shocks

**Fig. 5:**
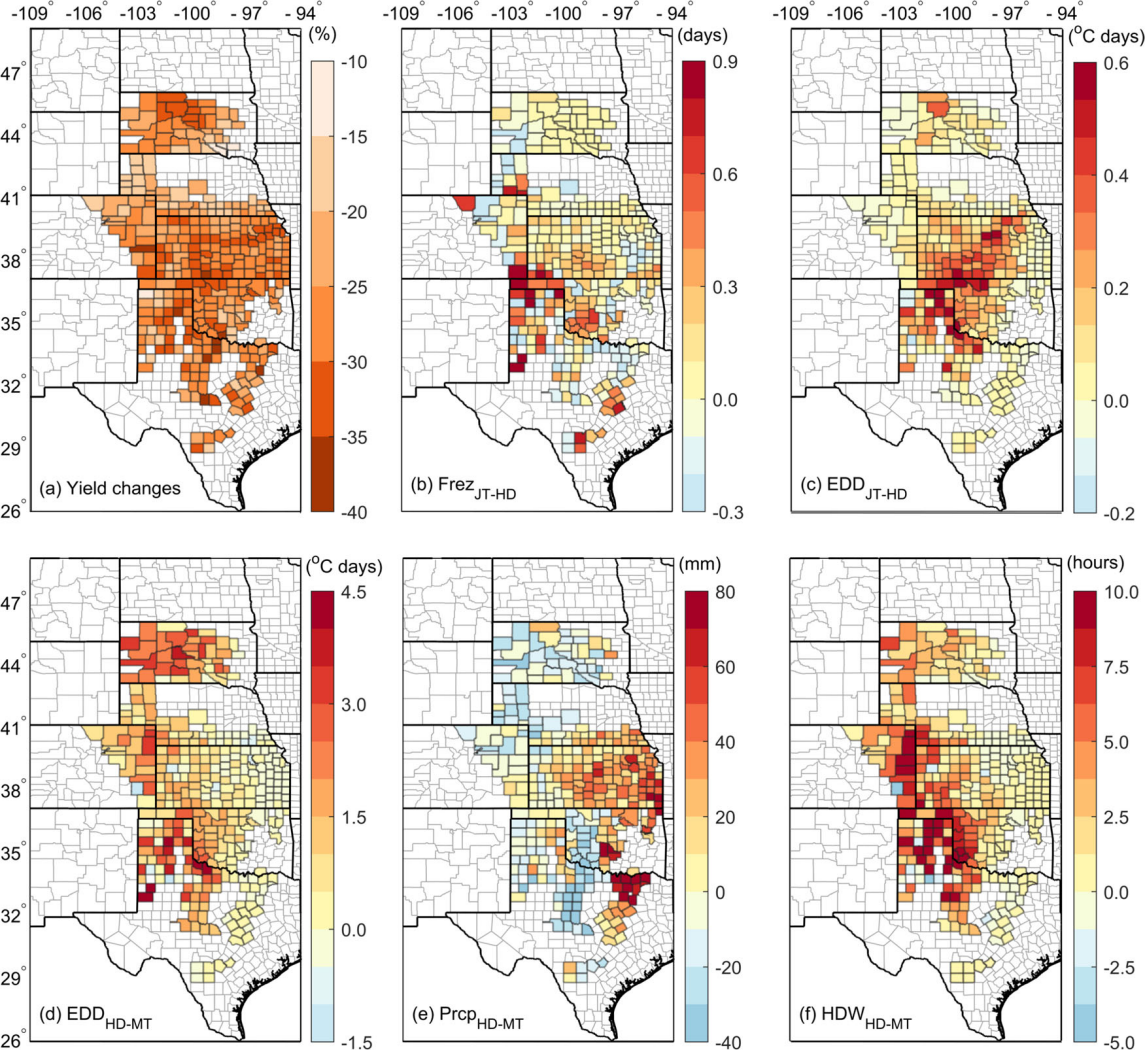
Yield shock distributions and a decomposition of yield shock drivers. **a** Spatial distribution of the county**-**level yield shock. **b**–**f** The concurrent changes in climate indices including freezing days (Frez, (**b**)), extreme degree days (EDD, (**c**)) from the jointing (JT) to heading (HD) stage, as well as including EDD (**d**), precipitation (Prcp, (**e**)), and hot-dry-windy (HDW, (**f**)) from the heading (HD) to maturity (MT).

To further examine climate impacts (Fig. 2) using spatial patterns of yield loss, we studied the anomalous behavior of climate indices during yield shock years. We defined a “yield shock” year as a year in which the yield change percentage from a five-year moving average fell below each county’s 25th percentile from 1982 to 2020 inclusive (Fig. 5a) (see Methods for details). The yield shocks could accordingly be attributed to multiple causative climate indices. Specifically, when a yield shock occurred, we found the main climate indices corresponding with anomalous wheat yields were EDD_JT-HD_ in the central winter wheat belt, diagonally distributed from the panhandles of OK and TX to northeastern KS, and EDD_HD-MT_ in the western areas. Both of these factors impaired wheat physiological processes critical to reproduction^46^. Excessive Prcp_HD-MT_ that occurred in the eastern U.S. Southern Great Plains^47^ are associated with yield reductions via soil moisture saturation, waterlogging, disease exposure, and lodging^48^. Additionally, drought conditions (lower precipitation) are associated with reduced yields in the western and northern parts of the study domain. The most HDW affected areas corresponding to the yield shocks were located in the western U.S. hard winter wheat belt within the same footprint as the 1930s Dust Bowl^17,20^ (Figs. 1 and 5f). These anomalous climate indices are most likely the main yield shock drivers across the states and sub-regions, and they support our findings drawn from statistical modeling (Fig. 2a, b) and provide the spatial sensitivities of winter wheat yields with respect to climate indices including compound HDW events (Fig. 5).

In this study, we focused on the impacts of HDW along with other main climate indices (Frez, EDD, and Prcp) on winter wheat yields. The mechanism of compound HDW exposure, like other climate compound events^11,12,18^, is shaped by extreme climate drivers, agronomic management, and cropping forces, which cause their impacts to be amplified compared to the impacts from those same events (Fig. 2c) occurring in a univariate way^11,12^. Crop vulnerability under HDW exposure can be complicated depending on the wheat varieties not only due to varying phenology, but also inherent genetic heat stress tolerance, water management, and the interaction of other hazards such as pests and diseases at various climate and agronomic scales. This is particularly true for winter wheat systems, given the long growing season that spans three distinct calendar seasons (fall, winter, and spring). To possibly mitigate HDW impacts on wheat yields, the observed HDW trends and impacts in the context of climate change underscore the need to adapt crop management and genetics to concurrent hot, dry, and windy conditions. The likelihood of the continuation of HDW in the U.S. Great Plains might be ‘atmospheric-bridged’ by the PDO variations. Unlike El Niño and La Niña events (an inter-annual scale), the PDO temporal evolution is marked by a variability of ~50–70 year periodicity^49^. Cropping adaptations might avoid antagonisms among individual stress processes considering compound HDW events could ramp up in the future, especially when their occurrence is modulated with locations (climate), wheat varieties (genetics), and crop management conventions (water and chemical management by local or regional farmers). Thus, further studies are needed to better understand the relationship between compound extreme events and crop yield, including evaluating impacts directly associated with changes of frequency and intensity (i.e., amplitudes) of HDW occurrence during the grain-filling period in the U.S. wheat belt. The exploration of the HDW-yield relationship in this study is a first step towards understanding the impacts of potential compound climate extreme events on crop yields, because HDW involves the co-occurrence of three, dependent hazards: unusual heat, drought, and high winds. Our results showed that compound HDW are associated with negative impacts at a scale well beyond which any one of them, being in an extreme state, might have caused on its own.

In summary, the severe HDW-affected areas over the past 39 years were similar to the areas affected by the Dust Bowl of the 1930s, and HDW trends during the HD-MT period significantly increased in the U.S. hard winter wheat belt. HDW during the HD-MT stage was the most influential climate index affecting winter wheat yields, and winter wheat productivity has apparently been reduced by increased HDW events during the last 39 years. The statistical significance of climate extreme indices coincided with the spatial patterns of hard winter wheat yield shocks in the U.S. winter wheat belt. Our results highlight the fact that climate change can significantly impact winter wheat production not only through changes or distribution shifts from single-variable climate extremes such as heat stress, drought events, and cold damage but also more importantly through climate change-related compound climate events, which amplified the impacts on wheat yields. In the context of climate change adaptation, our study shows that the combination of hot, dry, and windy conditions is an under-appreciated risk to food production in the U.S. as well as in the world’s winter wheat production areas.

## Methods

### Winter wheat county selection

To ensure robust analysis and modeling, county**-**level yields were collected only for those counties with >5000 acres of harvested hard winter wheat and at least 20 years of available yield data. Accordingly, yield data from 339 counties across six states, i.e., South Dakota (SD), Nebraska (NE), Colorado (CO), Kansas (KS), Oklahoma (OK), and Texas (TX), were used in the study. The county**-**level hard winter wheat yield data between 1982 and 2020 were obtained from information provided by the United States Department of Agriculture’s National Agricultural Statistics Service (USDA**-**NASS). All yield data in this study were directly taken from survey yields, and the irrigation fraction was <5% on average in our study domain^19^.

### Climate

Hourly climate data of 2**-**m air temperatures (T, °C) and relative humidity (RH, %), computed from dew point temperature^50^, and 10**-**m wind speed (U, m s^−1^) were obtained from the ERA5**-**Land dataset from 1982 to 2020. The ERA5**-**Land is a global atmospheric reanalysis product produced by the European Centre for Medium**-**Range Weather Forecasts (ECMWF)^51^. The Centre recently has significantly improved the accuracy of the data for all types of land surface applications. Daily precipitation (mm) data were obtained from the Parameter-elevation Regressions on Independent Slopes Model (PRISM) data, which was developed based on observing networks and was commonly used to assess the relationship between precipitation and crop growth and development^52,53^. The county**-**level climate data were from the nearest grid points to the county centroid. We also used quality-assured station observations from the Met Office Hadley Centre’s global sub-daily station dataset (HadISD)^54^ to verify the spatial distribution of annual HDW during the heading to maturity stage (Fig. 1 and Supplementary Text 2). The climate dataset sources are listed in Supplementary Table 4.

### Phenology

The phenological data included planting dates from 230 experimental stations and harvesting dates from 186 experimental stations (Supplementary Fig. 2a, d). There were three stations in Texas documenting the heading dates (Supplementary Fig. 2c). The maturity dates were inferred from dates 14 days prior to harvesting dates^55^ for all county-years. Wheat jointing and heading dates were estimated using the accumulated GDD required for winter wheat maturity^56^. In each county**-**year combination, we partitioned the life cycle of winter wheat into three phenological stages: planting to jointing (PT**-**JT), jointing to heading (JT**-**HD), and heading to maturity (HD**-**MT) (Supplementary Tables 1 and 5; Supplementary Text 1; Supplementary Figs. 1 and 2).

### Model development

We used a linear mixed-effects model to examine the impacts of climate indices including HDW (hours), freezing days (Frez, days), extreme degree days (EDD, °C days), and precipitation (Prcp, mm) (Supplementary Fig. 1). The model with site- and year- fixed effects was used to estimate the impacts of climate indices on yields:

1 $${Y}_{i,t}={({f{W}_{i,t}{{{{{\rm{;}}}}}}\beta }_{{env}})+{c}_{i}+{y}_{t}+\varepsilon }_{i,t}$$

where *Y*_i,t_ is the yield at county *i* in year *t*. The *f(W*_*i,t*_*; β*_*env*_*)* represents the effects of county**-**specific weather (*W*_*i,t*_) in either standard units or original units on yields. Two control predictors are a county-specific vector (*c*_*i*_) to account for time-invariant spatial heterogeneity and a time-fixed effect vector (*y*_*t*_) to control yield improvements and/or changes in crop breeding technologies and innovations in crop production practices over time (Supplementary Fig. 8). *ε*_*i,t*_ is the random error term. The error terms *ε*_*i,t*_ might be heteroskedastic and autocorrelated in year and county domains. Therefore, two-way clustering standard errors (SEs)^57^ were implemented to consider the effects of heteroskedastic and autocorrelation in estimating coefficients in Eq. (1). This adjusted standard error leads to more conservative adjusted *p*-values (*p*_adj_) (Supplementary Tables 2 and 3) that we used. Models that logarithmically transformed yields were also tested, and all the results were similar or consistent when using logarithm yields rather than original yields. Our preferred specification used yields because this model had more explanatory power and more normally distributed residuals.

To directly compare the yield sensitivity to climate indices, we standardized the original climate data as “standardized anomaly” (also known as z**-**score) by subtracting the mean and then dividing by one standard deviation. This transformation achieved the standardized regression coefficients and can be used to explain sensitivities with respect to individual climate indices (referred to as ‘per standard unit’)^58^, and is also widely used to assess the impacts of excessive rainfall on maize yields^59^. In addition to the modeling with standardized predictors (Fig. 2a and Supplementary Table 2), climate indices without any data transformation were also used to fit the model for straightforward interpretation on their natural scales (Supplementary Table 3).

### Yield sensitivity

The yield sensitivity was reported based on phenological stages (p = 1, 2, and 3 for PT**-**JT, JT**-**HD, and HD**-**MT stages, respectively) (Supplementary Figs. 1 and 2) for each county**-**year, representing a life cycle of winter wheat. The weather function is defined as follows:

2 $${f\big({W}_{i,t}{{{{{\rm{;}}}}}}\,\beta }_{{env}}\big)=	 \mathop{\sum }\limits_{p=1}^{2}{\beta }_{1,p}{{Frez}}_{i,p,t}+\mathop{\sum }\limits_{p=2}^{3}{\beta }_{2,p}{{EDD}}_{i,p,t}\\ 	+\mathop{\sum }\limits_{p=1}^{3}\left({\beta }_{3,p}{{Prcp}}_{i,p,t}+{\beta }_{4,p}{{{Prcp}}_{i,p,t}}^{2}\right){+{\beta }_{5}{HDW}}_{i,t}$$

where *Frez* measured the exposure days where the minimum temperatures were below the thresholds^38^. *EDD* referred to the accumulation of degree days over 32 °C^60^, and *Prcp*_*i,p,t*_ and *Prcp*_*i,p,t*_^*2*^ accounts for a quadratic polynomial effect for the cumulative precipitation during the phenological phase p. *HDW* is accumulated over the HD-MT period. Frez_HD**-**MT_, EDD_PT**-**JT_, HDW_PT-JT_, and HDW_JT-HD_ were not included in the model due to their infrequent occurrence under actual conditions (<5% of the year-county combination). The thresholds selected for specific phenological stages are detailed in Supplementary Fig. 1. Additionally, we further expanded the model (Eq. 2) to assess the impact of HDW occurrence on wheat yields during the specific sub-stage of heading to maturity. We partitioned the heading-maturity stage into three sub-stages (i.e., early, middle, and last sub-stages) and statistically estimated the sub-stage specific HDW influence on yields.

Whether or not the compound HDW events amplified the impacts on wheat yields during the HD-MT stage motivated us to compare impacts of individual and compound extreme events on wheat yields. we re-analyzed extreme climate events into individual, bivariate, and trivariate compound events: (1) only hot [H: T ≥ 32 °C but (RH > 30% & U < 7 m s^−1^)]; (2) only dry [D: RH ≤ 30% but (T < 32 °C & U < 7 m s^−1^)]; (3) only high winds [W: U ≥ 7 m s^−1^ but (T < 32 °C & RH > 30%)]; (4) compound hot-dry [H&D: (T ≥ 32 °C & RH ≤ 30%) but U < 7 m s^−1^]; (5) compound hot-winds [H&W; (T ≥ 32 °C & U ≥ 7 m s^−1^) but RH > 30%]; (6) compound dry-winds [D&W; (RH ≤ 30% & U ≥ 7 m s^−1^) but T < 32 °C]; and (7) compound hot-dry-windy [HDW: T ≥ 32 °C & RH ≤ 30% & U ≥ 7 m s^−1^]. These classified events are treated separately so that we could evaluate their individual impacts within our statistical model (Eqs. 1 and 2). The impact of climate indices on yields (Fig. 3) were quantified^61,62^ by multiplying the yield sensitivity with respect to climate indices in original units (Supplementary Table 3) and the trend values of corresponding climate indices (Supplementary Fig. 9e–h).

### Statistical analysis

All trend values in this study were estimated by ordinary least-square regression, but the statistical significance was tested by Mann**-**Kendal analysis throughout our study at a 95% confidence level^63^ or otherwise indicated. The Mann-Kendal method can accommodate data that are not normally distributed, and is insensitive to outliers. Because the HDW could be viewed as count data, as an alternative, Poisson regression^63^ was also used in the HDW trend analysis. The results showed that the *p*-values from Poisson regression were consistent with the Mann–Kendal method and their trend values were also consistent with the least-square regression (Supplementary Fig. 6). Finally, linear correlation measures (both the Pearson and Spearman methods) were used to evaluate correlations at the 95% confidence level. We analyzed temporal-spatial correlation between PDO and HDW with 70-year data (1951–2020). To minimize the climate change effects on correlations between PDO and HDW, we used partial Spearman’s rank correlation to estimate correlation between the PDO and HDW anomaly by controlling mean temperature (April-June) effects.

### Yield shock drivers

Relative yield change percentage (*Y’*) for county *i* in year *t* was calculated as,

3 $${{Y}^{{\prime} }}_{i,t}(\%)=\,\frac{{Y}_{i,t}-\,\frac{1}{5}\mathop{\sum }\nolimits_{i=t-2}^{i=t+2}{Y}_{i}}{\frac{1}{5}\mathop{\sum }\nolimits_{i=t-2}^{i=t+2}{Y}_{i}}\times 100$$

Here, *Y*′ is relative to the 5**-**year running mean yield (*Y*_*i*_), thus removing the interannual variation^19^. We then defined a yield shock year in each county as a year in which *Y’* fell below the 25th percentile over total yield years. The concurrent changes in climatic indices, calculated by the difference between the mean during yield shock years and the mean over the whole period (1982**–**2020) for each county (Fig. 5b–f), were considered potential climate drivers for yield shocks.

### Robustness checks

To consolidate our analysis, we conducted a series of robustness checks. We first re-calculated HDW events using a total of 36-threshold combinations: 4 temperatures [30, 31, 32, and 33 °C] × 3 relative humidity [25, 30 and 35%] × 3 wind speeds [6, 7, and 8 m s^−1^] (Supplementary Fig. 10). Then, we assessed the model (Eqs. 1 and 2) for each threshold combination. Results showed that the sensitivity of wheat yield to HDW events was −0.09 t ha^−1^ (10 h)^−1^ on the average of these 36 combinations, ranging from −0.06 to −0.13 t ha^−1^ (10 h)^−1^ (Supplementary Fig. 11). The averaged HDW effect is equal to the result we showed (Fig. 2a). This result indicates that the threshold we used can represent an averaged-level HDW effect.

To increase the confidence of HDW influence on wheat yields, we used both the quadratic temperature and the temperature bins’ model (Supplementary Text 4). We found that including temperature bins’ variables during three phenological stages provides an explicit nonlinear turning point of temperature (i.e., 27 °C during PT-JT, 29 °C during JT-HD, and 32 °C during HD-MT periods) but it did not offset the parameter estimate for HDW impacts in the model (Supplementary Fig. 12). Specifically, yield sensitivity derived from model of Eqs. 1 and 2 is slightly lower than the sensitivities in the quadratic temperature model and slightly larger than that in the temperature bins’ model (Supplementary Fig. 12b). Details associated with the robustness checks section are provided in Supplementary Text 4.

## Supplementary information

Supplementary Information

## Data Availability

The original data (phenological dates and yields of winter wheat) and underlying data have been deposited through a public repository at 10.6084/m9.figshare.19224693.v5. Climate data supporting the findings of this study are from publicly available datasets with specific links provided in the Supplementary Information.

## Code availability

The code used in this study to show main findings is available through a public repository at 10.6084/m9.figshare.19222548.v6. Other codes are available from the corresponding author upon request.
