# Supplementary material for: U.S. winter wheat yield loss attributed to compound hot-dry-windy events
Source: Nat Commun. 2022 Nov 24;13:7233. doi: 10.1038/s41467-022-34947-6 (PMC9700680; doi:10.1038/s41467-022-34947-6)
Supplement: Supplementary file 1 — Supplementary Information [file 41467_2022_34947_MOESM1_ESM.pdf]

Supplementary Information

U.S. winter wheat yield loss attributed to compound hot-dry-windy events

Haidong Zhao[1], Lina Zhang[2], M. B. Kirkham[1], Stephen M. Welch[1], John W. Nielsen-Gammon[3], Guihua Bai[4], Jiebo Luo[5], Daniel A. Andresen[6], Charles W. Rice[1], Nenghan Wan[1], Romulo P. Lollato[1], Dianfeng Zheng[7], Prasanna H. Gowda[8], Xiaomao Lin[1,2*]

[1]Department of Agronomy, Kansas State University, 2004 Throckmorton Hall, Plant Sciences Center, Manhattan, KS 66506, USA

[2]Kansas Climate Center, Kansas State University, 2108 Throckmorton Hall, Plant Sciences Center, Manhattan, KS 66506, USA

[3]Department of Atmospheric Sciences, Texas A&M University, College Station, TX 77843, USA

[4]Hard Winter Wheat Genetics Research Unit, USDA–ARS, Kansas State University, Manhattan, KS 66506, USA

[5]Department of Computer Science, University of Rochester, Rochester, NY 14627, USA

[6]Department of Computer Science, Kansas State University, Manhattan, KS 66506, USA

[7]College of Coastal Agriculture Sciences, Guangdong Ocean University, Zhanjiang, Guangdong 524088, China

[8]USDA, Agricultural Research Service, Southeast Area, Stoneville, MS 38776, USA

[*]**Corresponding author:** Xiaomao Lin (xlin@ksu.edu)

Contents

Supplementary Text 1. Phenology of winter wheat 3
Supplementary Text 2. HadISD data to identify HDW 4
Supplementary Text 3. Intercomparison between ERA5-Land and HadISD datasets 5
Supplementary Text 4. Additional statistical models tested 5
Supplementary Figures (12) 7
Supplementary Tables (5) 19

Supplementary Text 1. Phenology of winter wheat

Because weather varies in both space and time, uniform and homogenous responses of crops to weather during the growing season should not be expected. Thus, phenological dates for each county-year were estimated separately, which allows us to analyze yield responses physiologically in terms of climate change and variability that sub-regions (states or counties) were experiencing in a wheat life cycle (Supplementary Fig. 1). The phenological observations, including planting and harvest dates, were collected from 230 and 186 experimental stations, respectively (Supplementary Fig. 2a,d). Each state had more than 12 years of phenological observations with 15 stations per year on average. To fulfill a complete dataset, we first interpolated available data to the centroid of each county using a Delaunay Triangulation method[1]. Due to missing dates, especially in Oklahoma and Texas, we constructed linear regression models between phenological dates across Kansas (KS) and Nebraska (NE), where the data were nearly complete, and available phenological dates mostly located in Oklahoma (OK) and Texas (TX). We evaluated the completeness of our phenological date estimates by a leave-one-out approach[2]. The selection of the optimum coefficients for each model was based on the minimum root mean square error (RMSE) between fitted and observed phenological datasets (Supplementary Table 5). The selected model was then used to gap-fill missing planting and harvest dates for each county from 1982-2020. An empirical relationship, where physiological maturity occurred approximately 2 weeks prior to harvest[3], was used to estimate maturity dates in our study.

Growing degree days (GDD, ^{o}C days) is a common matrix used to partition phenological periods of winter wheat in the Great Plains[4]. For example, the jointing and heading dates were defined as reaching 35% and 57% of accumulated GDD[5] required for winter wheat maturity from January 1 to the maturity date, respectively[4]. Based on this averaged accumulated GDD,

the jointing and heading dates for all counties were estimated. Note that, even though there were no available documented harvest dates in Texas, the observed heading dates were collected over 20 years covering three counties including Bushland, Chillicothe, and Dallas in Texas, which represent three diverse climate sub-regions: Great Plains, North Central Plains, and Coastal Plains, respectively (Supplementary Fig. 2c). The accumulated GDD from January 1 to the heading dates were used to reversely estimate average accumulated GDD from January 1 to maturity dates across these three counties for each year. We then estimated the maturity dates in TX. Similar to SD, CO, and OK, the estimated jointing dates and heading dates were also obtained in TX. Finally, we partitioned the wheat phenology into three stages, including planting to jointing (PT-JT), jointing to heading (JT-HD), and heading to maturity (HD-MT) (Supplementary Figs. 1 and 2). All phenological data can be directly obtained from the links listed in Supplementary Table 1, but KS data from 1982-2007 were from printed documents that we digitized at the Department of Agronomy and Kansas Climate Center, Kansas State University.

Supplementary Text 2. HadISD data to identify HDW

The HadISD is a global sub-daily dataset based on the U.S. Integrated Surface Database (ISD) from NOAA's National Centers of Environmental Information (NCEI). The dataset covers 1931 to the present and is designed to study the long-term changes of extreme temperature, pressure, and humidity[6]. The station selection criteria were based on a suite of quality control tests, and data homogeneity assessments were conducted for air temperature, dew point temperature, sea-level pressure, and wind speed[6]. We retrieved observed hourly 2-m air temperature and air relative humidity and 10-m wind speed from 1982 to 2020. We screened 16 stations with less than 5% missing data for all three variables during the grain-filling periods, except for 4 stations in CO and OK that had less than 10% missing data. We then used

the regularized expectation maximization (rEM) procedures to refill data gaps[7]. Finally, we presented the spatial pattern of HDW (Fig. 1b) based on the HadISD dataset.

Supplementary Text 3. Intercomparison between ERA5-Land and HadISD datasets

To evaluate HDW variations derived from ERA5-Land datasets in both spatial and temporal domains, we calculated correlation coefficients between HadISD and ERA5-Land datasets. We screened 34 stations from HadISD datasets based on our data quality control procedure. Stations were selected with at least 30 years of data for analysis. A year was treated as missing in any station if this station contained more than 8% missing hourly data from April 1 to July 31st from 1982 to 2020, which generally covered the grain filling period (HD-MT) across our study domain. Outside of this window there were rare HDW events. Then we trimmed all ERA5-Land datasets to hourly, in order to match with the HadISD dataset that we selected (Supplementary Fig. 3). We then counted annual mean HDW events for each station-year by using HadISD and ERA5-Land datasets. The temporal HDW anomalies, calculated by taking all of 1982 to 2020 as the base period, were used to get a correlation coefficient between HadISD and ERA5-Land datasets for each station (Supplementary Fig. 4a). The spatial HDW anomalies were calculated based on all available stations for a year (Supplementary Fig. 4b). We found both temporal and spatial correlation coefficients of 0.8 in most stations and years (Supplementary Fig. 4a,b). Changes of HDW events between the latest 20 years and the early 19 years indicated there were increasing HDW events (Supplementary Fig. 4c), suggesting not only an increasing trend of HDW events in both datasets but also changes in magnitudes that were consistent in both datasets.

Supplementary Text 4. Additional statistical models tested

To increase the confidence of HDW influence on wheat yields, we used both the quadratic

temperature model (M1, Eq. S1) and the temperature bins' model (M2, Eq. S2), which are temperature orientated. Note that we did not include EDD and FDD when considering temperature as a predictor because the quadratic temperature and temperature bins have captured the extreme heat (EDD) and the extreme cold (FDD) effects[8]. Both the M1 and M2 structures are shown following:

$$Y_{i,t} = \sum_{p=1}^{3}\left(\beta_{1,p}T_{i,p,t} + \beta_{2,p}{T_{i,p,t}}^{2}\right) + \sum_{p=1}^{3}\left(\beta_{3,p}Prcp_{i,p,t} + \beta_{4,p}{Prcp_{i,p,t}}^{2}\right) + \beta_5 HDW_{i,t} + c_i + y_t + \varepsilon_{i,t} \quad [\mathrm{M1, Eq. S1}]$$

$$Y_{i,t} = \sum_{p=1}^{3}\int_{h}^{\overline{h}} g_p(h)\emptyset_{i,p,t}(h)dh + \sum_{p=1}^{3}\left(\beta_{1,p}Prcp_{i,p,t} + \beta_{2,p}{Prcp_{i,p,t}}^{2}\right) + \beta_3 HDW_{i,t} + c_i + y_t + \varepsilon_{i,t} \quad [\mathrm{M2, Eq. S2}]$$

where T refers to mean temperature during specific phenological stage. The observed temperatures (h) during each time period range between the lower bound h and the upper bound $\overline{h}$. The *g(h)* is the fixed coefficient function for the temperature exposure, h, on yield. The $\emptyset_{i,p,t}(h)$ is the time distribution given the hours of exposure to temperature h. Other factors are the same using model Eqs. 1 and 2 as in the Method section of Main text. We here used temperature bins of 2°C interval during three phenological stages.

Supplementary Figures (12)

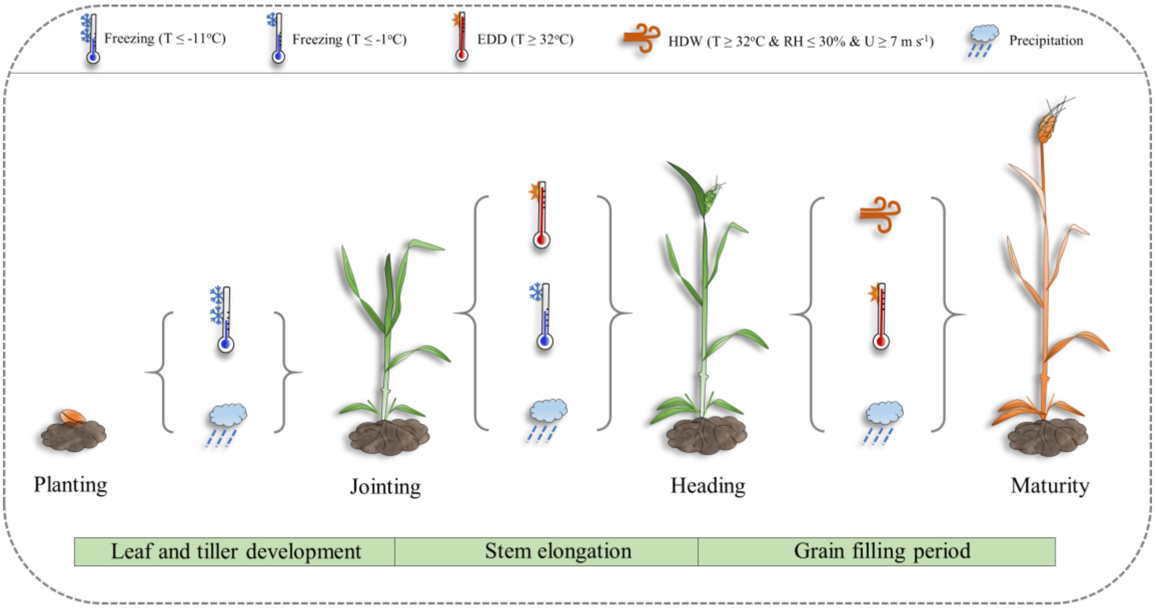

Supplementary Figure 1. Flowchart of main climate indices throughout three phenological stages in winter wheat. The thresholds selected (at the top) and three phenological stages (at the bottom) were used in our modeling process (at the middle, for example, we used EDD (extreme degree days), Freezing at T ≤ -1ºC, and precipitation during the jointing to heading stage. The planting-jointing (PT-JT), jointing-heading (JT-HD), and heading-maturity (HD-MT) stages were included during modeling.

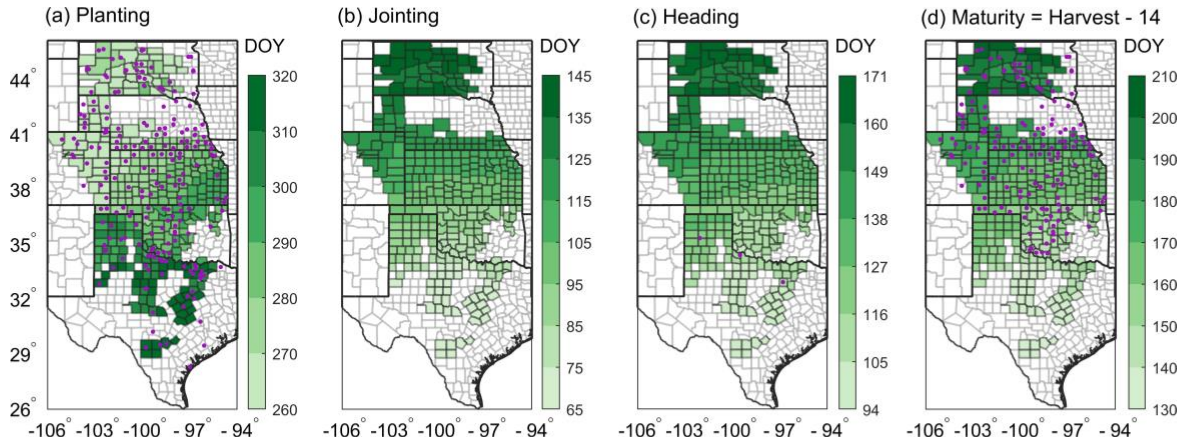

Supplementary Figure 2. Average phenology (days of year, DOY) estimated from 1982-2020. The estimated county-level phenology included **(a)** planting, **(b)** jointing, **(c)** heading, and **(d)** maturity dates. The purple dots in **(a)**, **(c)**, and **(d)** indicate the 230, 3, and 186 field experiment stations respectively, where planting, heading, and harvest dates were documented by observations (details in Supplementary Text 1).

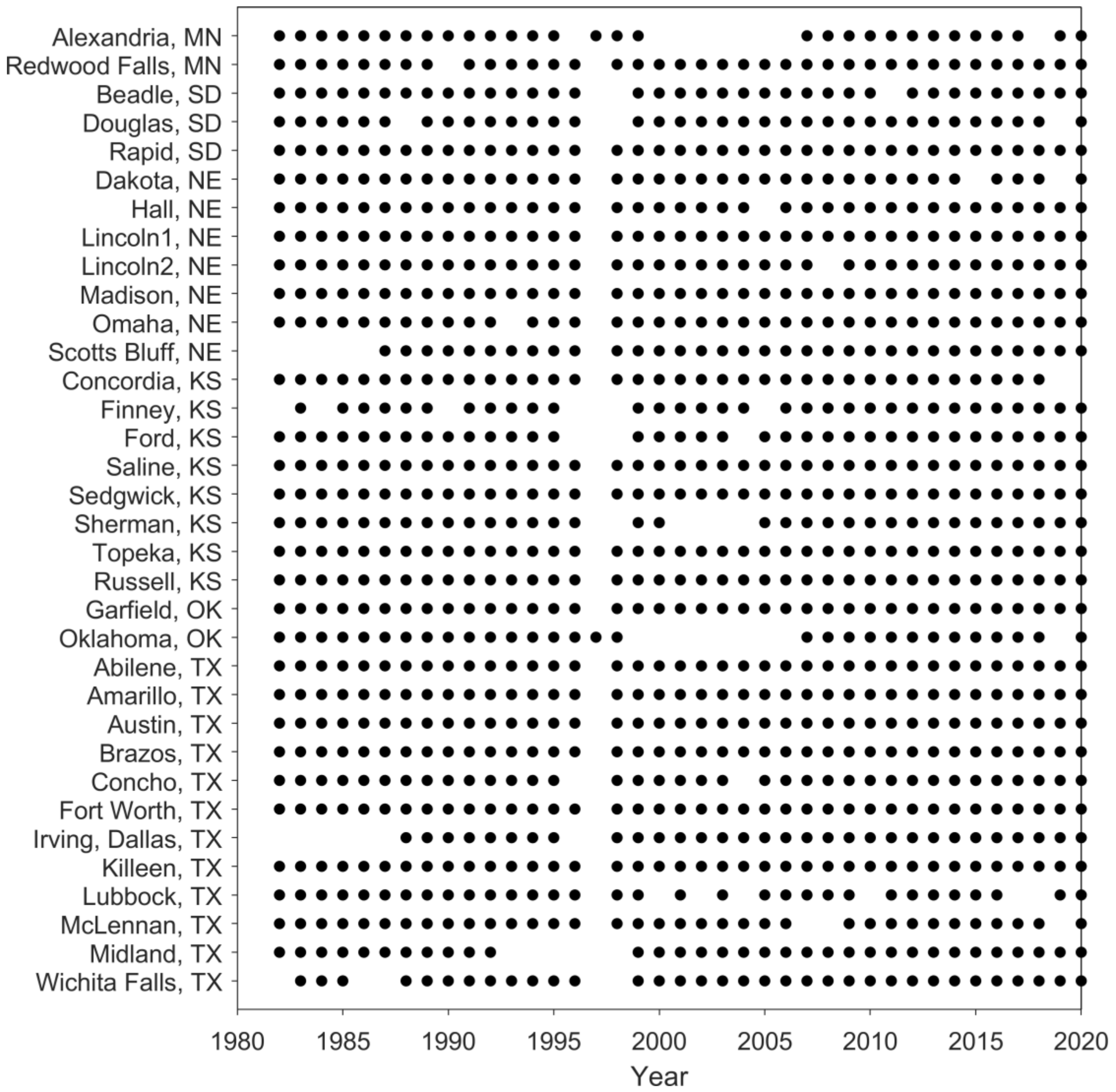

137 **Supplementary Figure 3. Available stations and years (trimmed from April 1 to July 31**
138 **only) for hourly temperature, relative humidity (dew point temperatures), and wind**
139 **speeds.** The dots represent observations available in years.

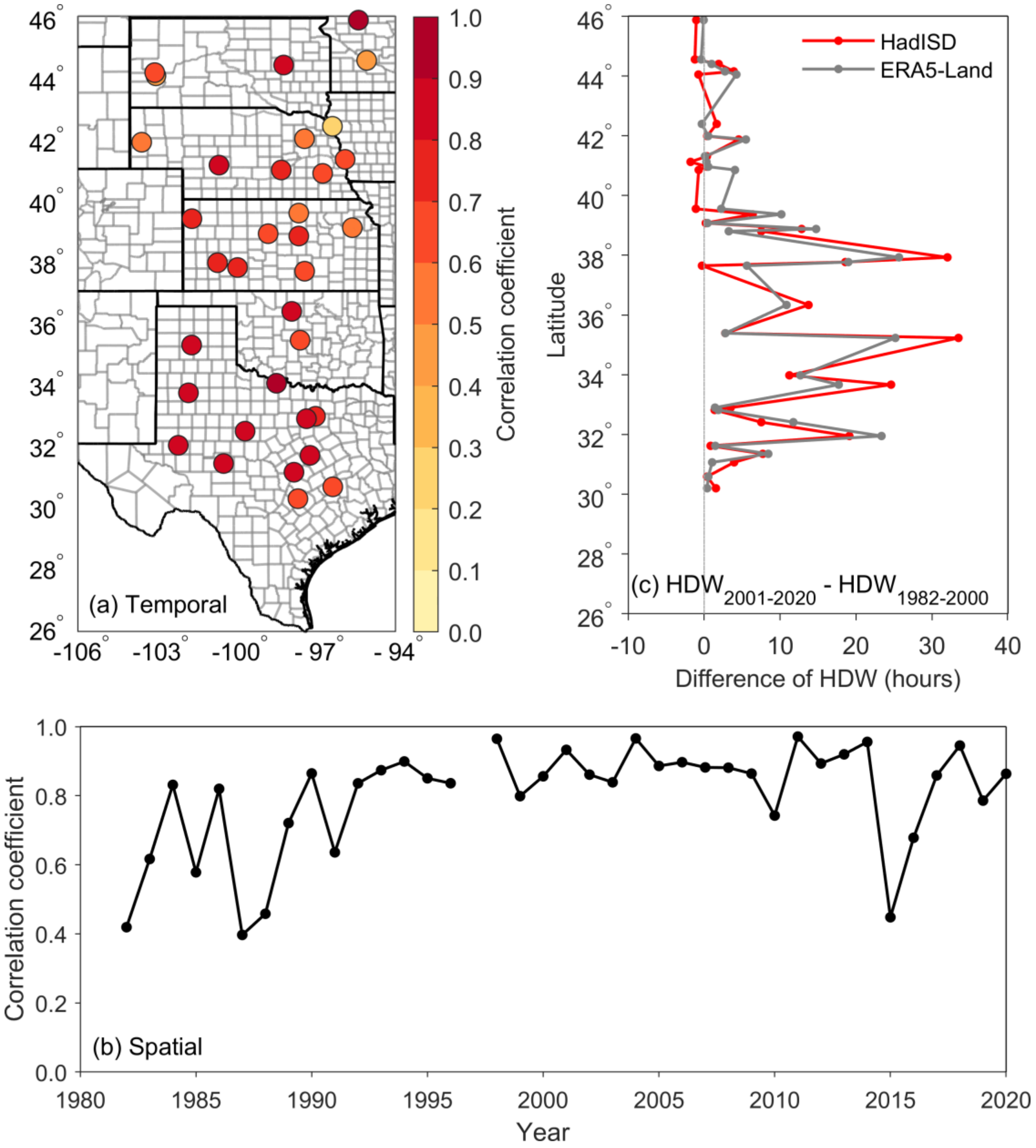

Supplementary Figure 4. The 34-station (a) temporal and (b) 39-year spatial correlation coefficients as well as (c) difference of averaged hot-dry-windy (HDW) events for the latest 20 years (2001-2020) and the earliest 19 years (1982-2000). All correlations calculated were based on either a temporal anomaly (average of available years as a base for a station) or a spatial anomaly (average of all available stations as a base for a year) from HadISD stations and ERA5-Land datasets.

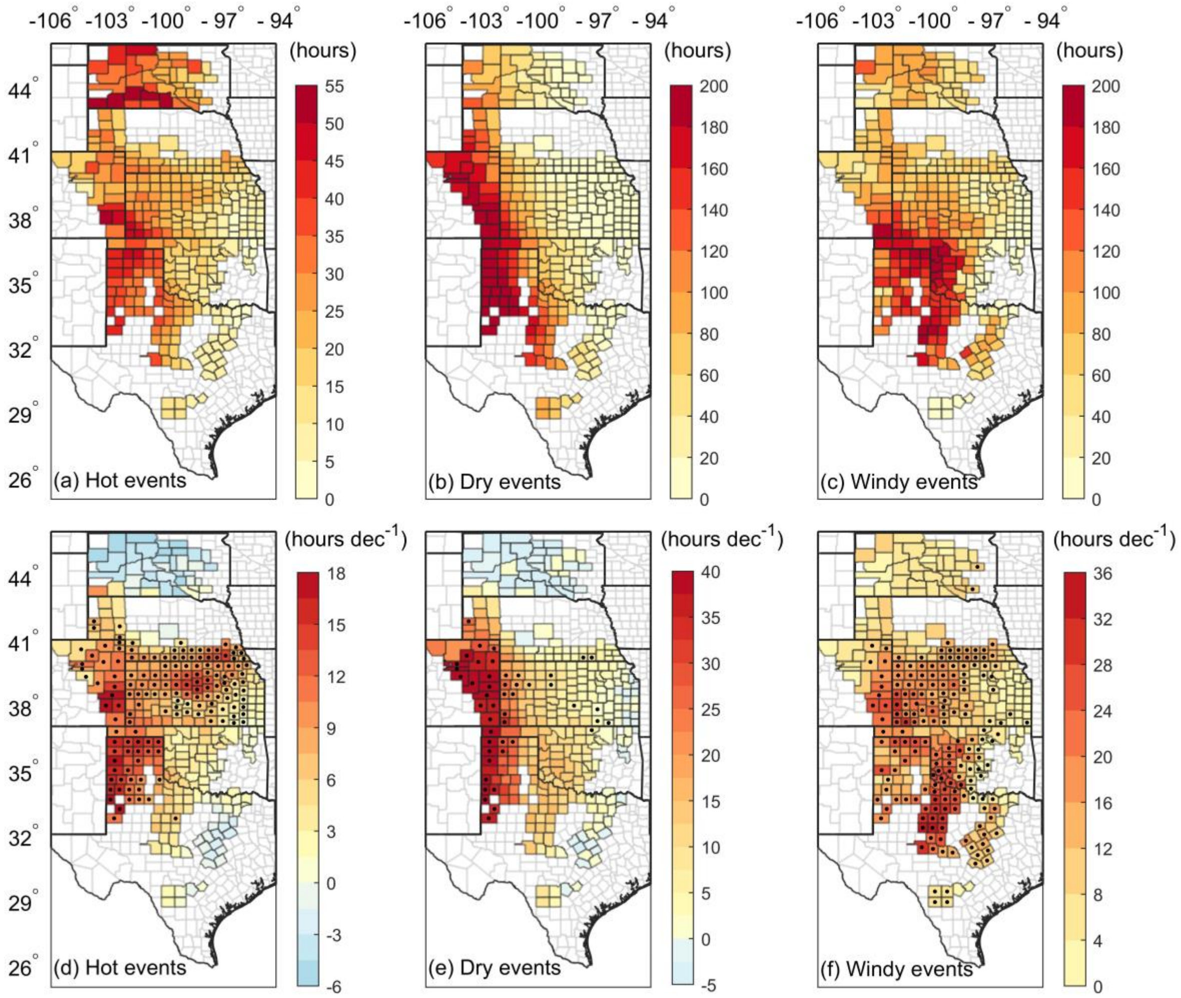

Supplementary Figure 5. Annual mean (a-c) and trends (d-f) of three individual climate extreme events during the heading-maturity (HD-MT) stage from 1982 to 2020. The annual mean of **(a)** hourly hot ($T \geq 32^{o}C$), **(b)** dry ($RH \leq 30\%$), and **(c)** windy ($U \geq 7$ m s^{-1}) events during HD-MT. The black dots indicate statistically significant trends at a 95% confidence interval based on the Mann-Kendall test ($p \leq 0.05$).

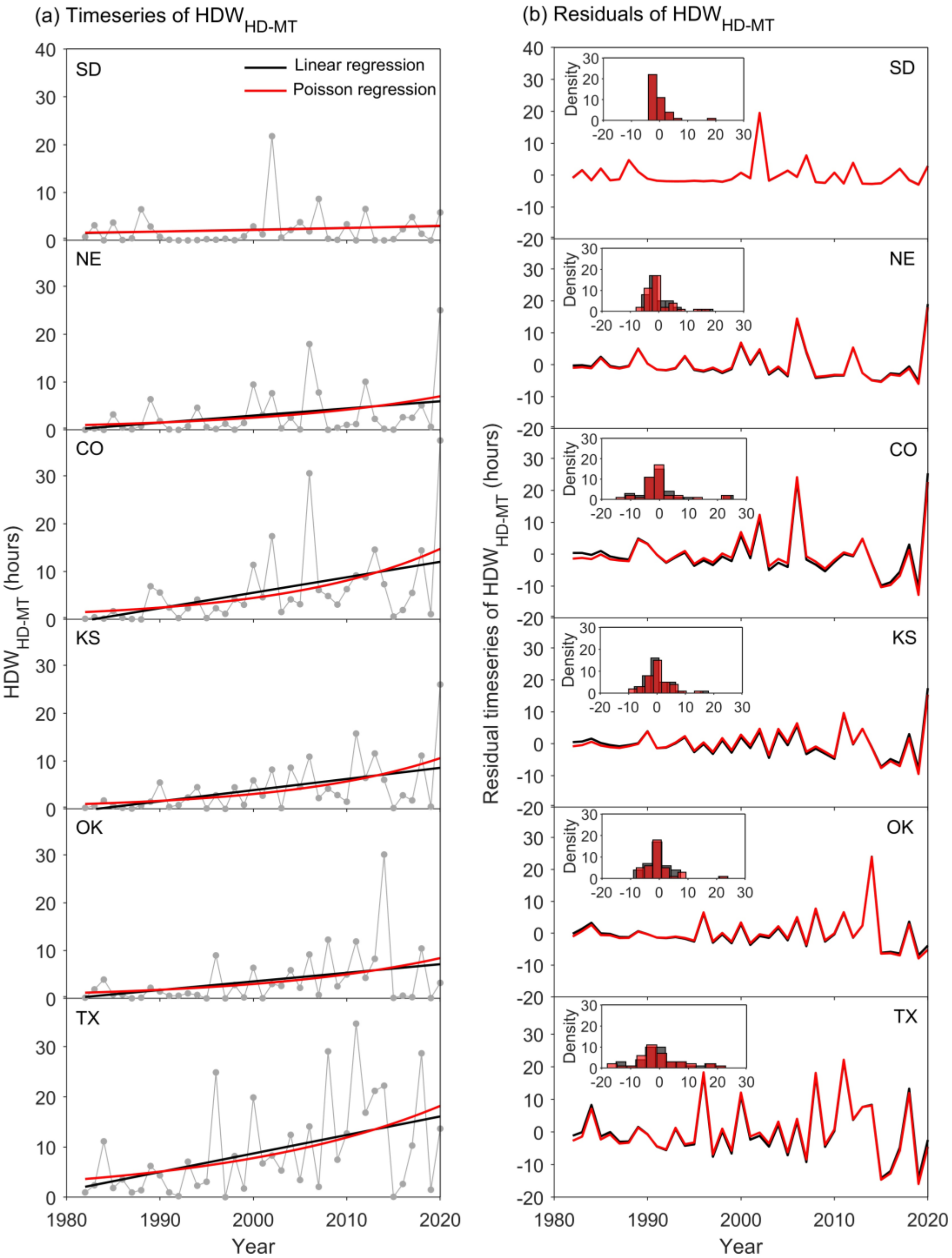

Supplementary Figure 6. Statewide hot-dry-windy (HDW) trends during the heading-maturity ($HDW_{HD\text{-}MT}$) stage when area-weighted averages were conducted from 1982 to 2020. a, Trends of $HDW_{HD\text{-}MT}$ detected by ordinary linear regression and Poisson regression, which should not be extrapolated because trend analyses (including other methods not used here), on average, best represent an approximation. **b**, residuals and their histograms.

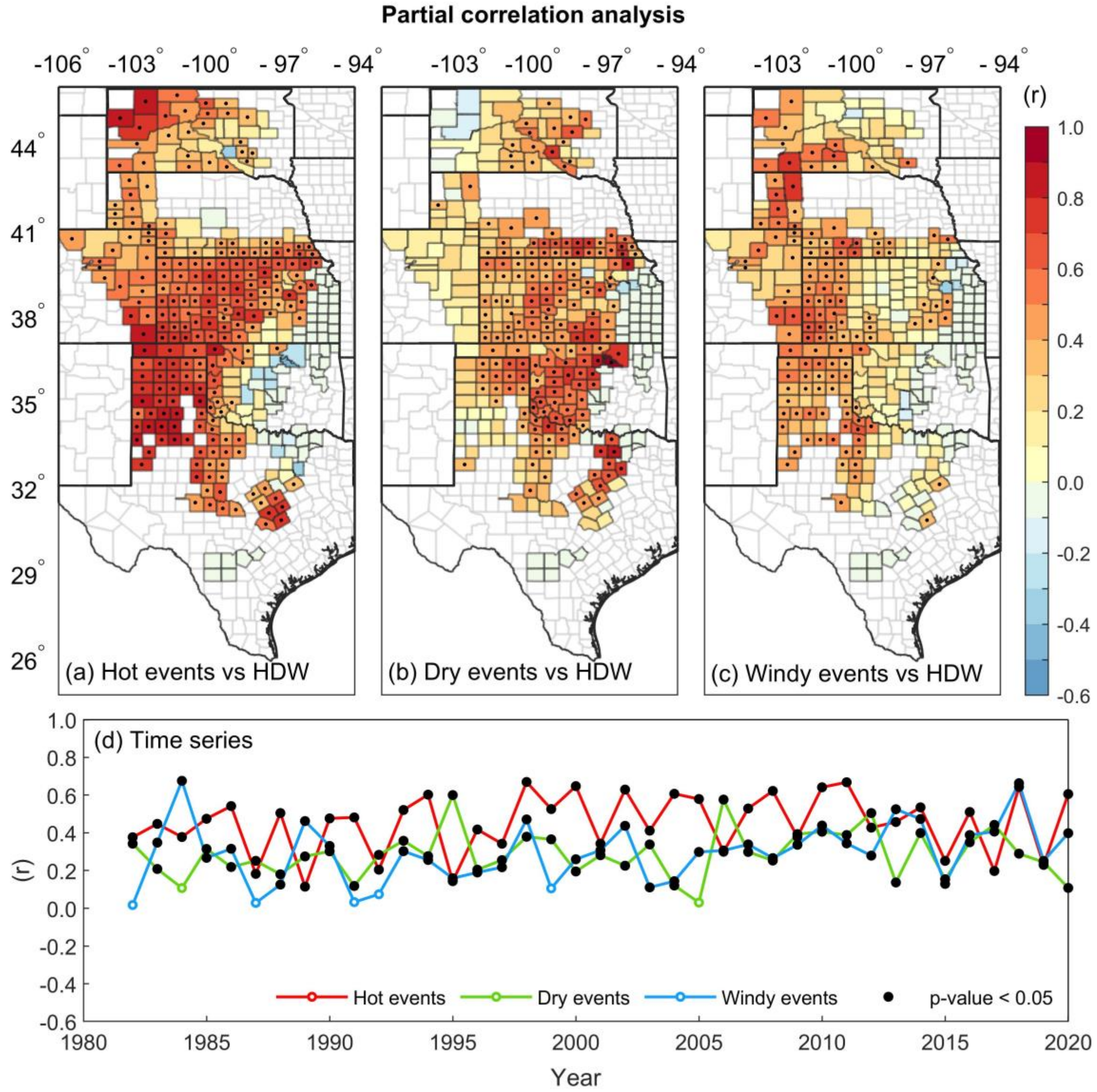

Supplementary Figure 7. Spatial (a-c) and temporal (d) patterns of partial correlation coefficients between three individual events and HDW during the heading-maturity (HD-MT) stage from 1982 to 2020. The black dots represent the statistically significant correlation years ($p \leq 0.05$).

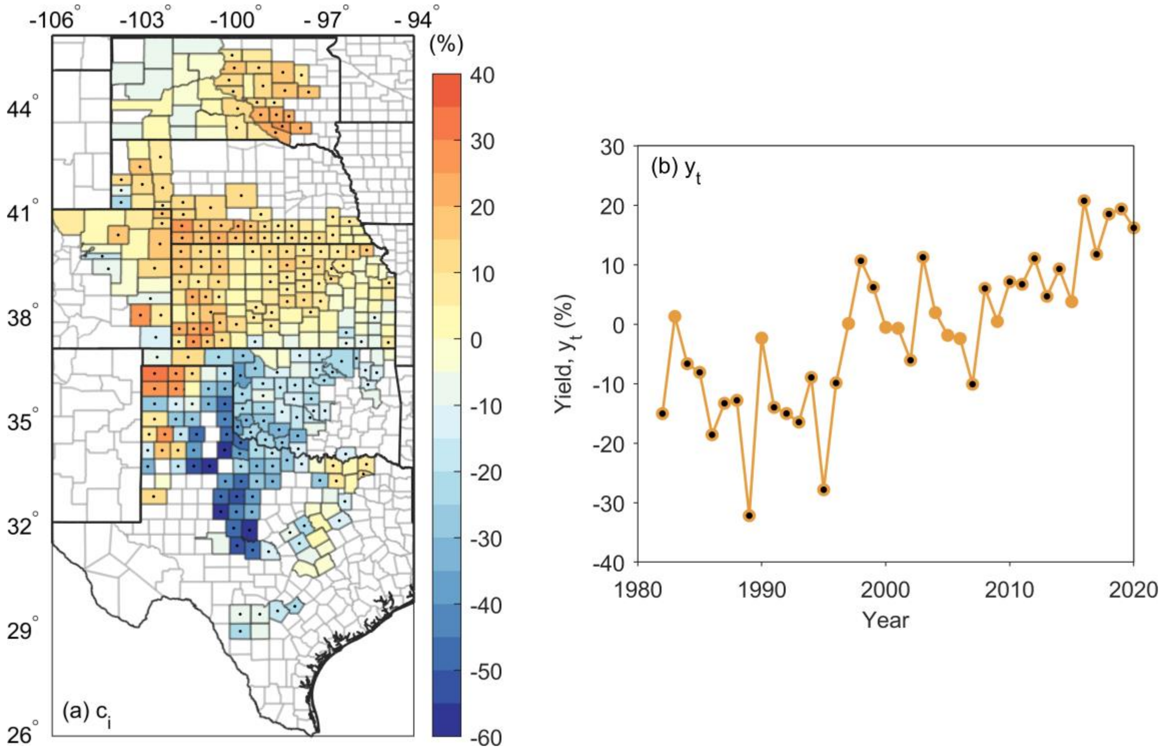

Supplementary Figure 8. The random effect of counties and years derived from the linear mixed-effects model relative to averaged yields (%). a, c_i term in Eq. (1). The color bar shows the effect sizes. **b**, y_t term in Eq. (1) showing a general upward trend whose overall magnitude is consistent with the rates of wheat breeding progress and improvements in agronomic production practices. The black dots indicate statistical significance in the model ($p \leq 0.05$).

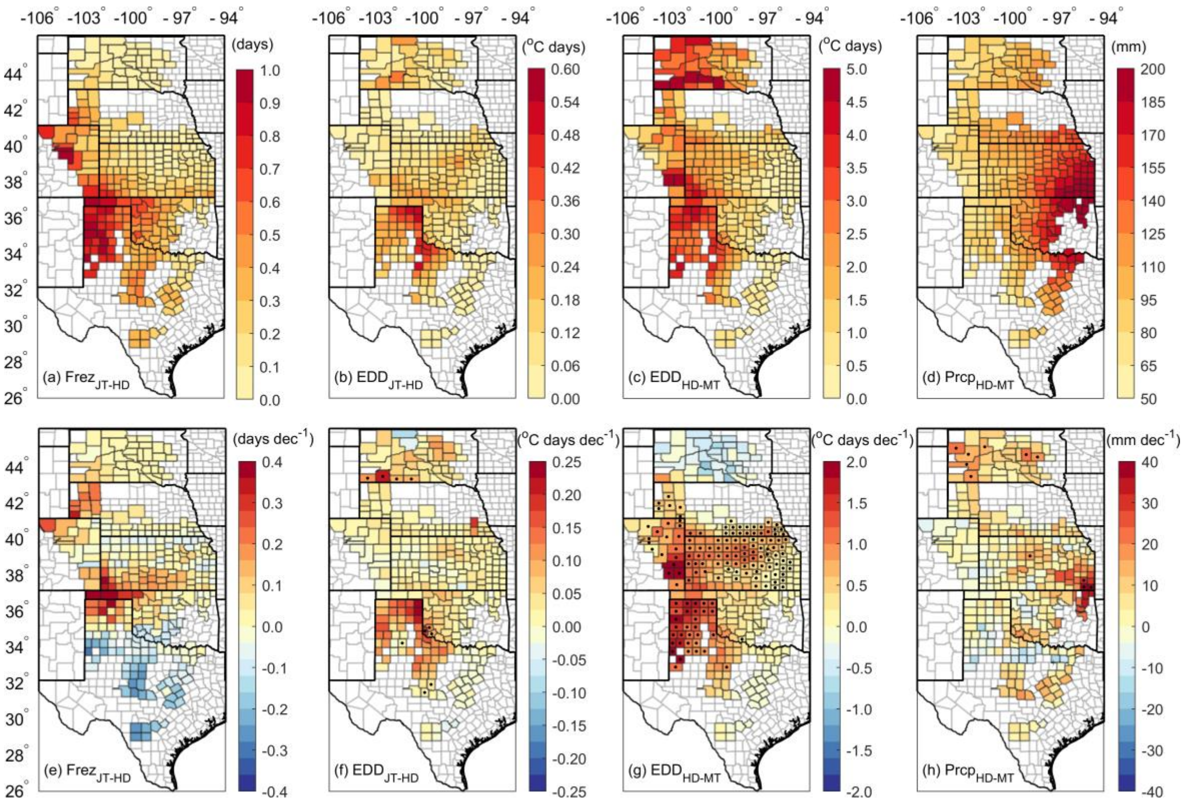

Supplementary Figure 9. Annual mean (a-d) and trends (e-h) of climate indices from 1982 to 2020. The black dots in **(e-h)** represent trends that were statistically significant in these counties ($p \leq 0.05$).

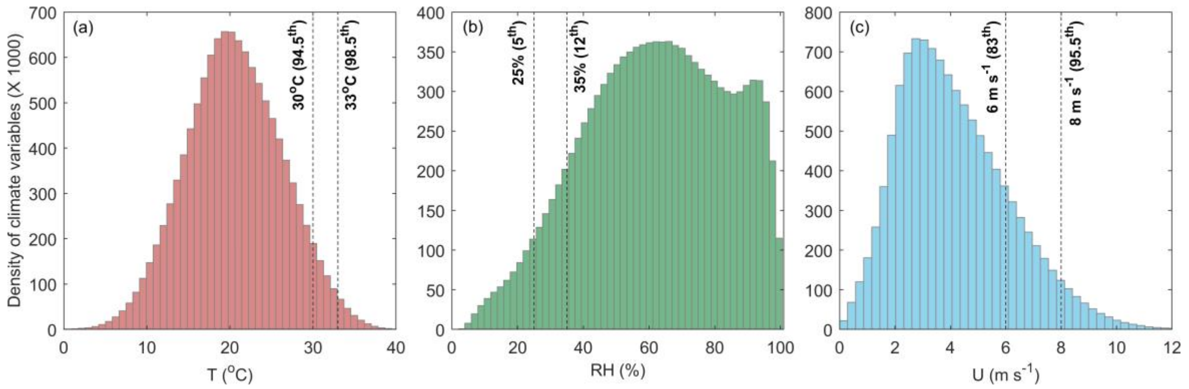

Supplementary Figure 10. **Distributions of (a) hourly temperature (T, °C), (b) relative humidity (RH, %), and (c) ambient wind speeds (U, m s^{-1}) during the heading to maturity period across our study domain from 1982-2020.** The black vertical dotted lines indicate the percentiles in brackets and their corresponding values.

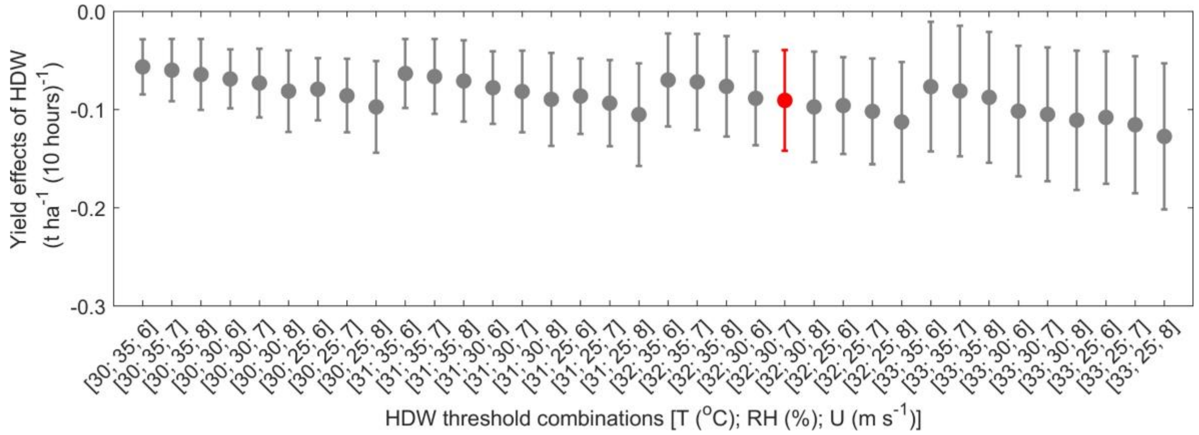

Supplementary Figure 11. **Yield sensitivity of hot-dry-windy (HDW) events from 1982 to 2020 estimated using the 36 combinations of the thresholds tested.** Red color indicates the combination we used in the Main text. Numbers in the square brackets represent thresholds of temperature (T), relative humidity (RH), and wind speeds (U), respectively. The error bars indicate a 95% confidence interval.

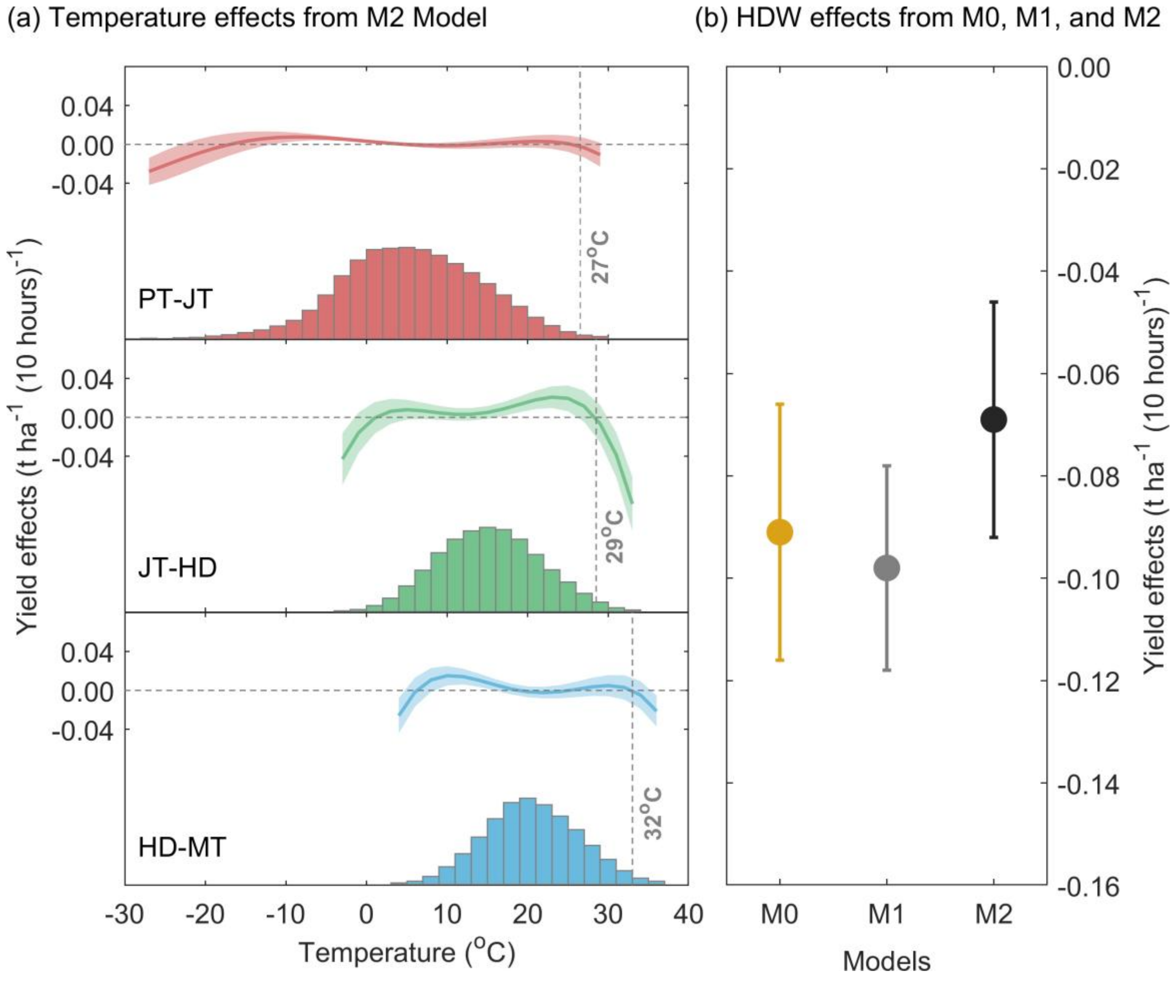

Supplementary Figure 12. Yield sensitivity to temperature bins and hot-dry-windy (HDW) effects tested in three models. a, Yield sensitivity to temperature bins at three phenological stages: planting to jointing (PT-JT; red); jointing to heading (JT-HD; green); and heading to maturity (HD-MT; blue). Histograms show exposure times (hours) for each phenological stage across county-years in 2°C temperature bins. The solid lines indicate the mean yield effects per 10 hours of temperature bin, and the shaded regions around the solid lines indicate the 95% confidence interval. Temperature "32°C" in the panel (blue) indicates that yield would be lost when temperature is higher than this threshold. **b**, Yield effects (t ha^{-1} per 10 hours of HDW events) during the HD-MT stage using the original model (here called M0, Eqs. 1 and 2 in Methods), the quadratic temperature model (M1, Eq. S1), and the temperature bins' model (M2, Eq. S2). Error bars show one clustering standard error (SE).

Supplementary Tables (5)

Supplementary Table 1. Links of publicly available data for phenology.

States	Links
South Dakota	https://extension.sdstate.edu/winter-wheat-variety-trial-results
Nebraska	https://cropwatch.unl.edu/winter-wheat-variety-test-results<br>https://digitalcommons.unl.edu/extensionhist/
Colorado	https://agsci.colostate.edu/csucrops/winter-wheat-trial-results/#Wheat2011
Kansas	https://www.agronomy.k-state.edu/services/crop-performance-tests/winter-wheat/index.html
Oklahoma	http://croptrials.okstate.edu/wheat/grain-yield/<br>https://digitalprairie.ok.gov/digital/collection/stgovpub/id/23993<br>https://www.okwheat.org/research-documents/variety-trials/
Texas	http://varietytesting.tamu.edu/wheat/<br>https://www.ars.usda.gov/plains-area/lincoln-ne/wheat-sorghum-and-forage-research/docs/hard-winter-wheat-regional-nursery-program/pre-2010-nursery-data/

Supplementary Table 2. Parameter estimates from standardized modelling regression. Standard errors (SEs) were clustered by county and year.

Climate indices	β	SEs	p_{adj}
$Frez_{PT\text{-}JT}$	-0.026	0.019	0.170
$Frez_{JT\text{-}HD}$	-0.029	0.011	0.009
$EDD_{JT\text{-}HD}$	-0.052	0.014	<0.001
$EDD_{HD\text{-}MT}$	-0.017	0.031	0.575
$Prcp_{PT\text{-}JT}$	0.500	0.051	<0.001
$Prcp_{JT\text{-}HD}$	0.190	0.044	<0.001
$Prcp_{HD\text{-}MT}$	0.062	0.043	0.144
$Prcp_{PT\text{-}JT}$ Squared	-0.383	0.051	<0.001
$Prcp_{JT\text{-}HD}$ Squared	-0.173	0.035	<0.001
$Prcp_{HD\text{-}MT}$ Squared	-0.118	0.040	0.003
$HDW_{HD\text{-}MT}$	-0.083	0.023	<0.001
R-squared	0.590		
p-value (F-test)	<0.001		

Supplementary Table 3. Parameter estimates from original modelling regression.

Climate indices	β	SEs	p_{adj}
$Frez_{PT-JT}$ (10 days)	-0.017	0.012	0.161
$Frez_{JT-HD}$ (1 day)	-0.033	0.013	0.013
EDD_{JT-HD} (1 ºC day)	-0.147	0.038	<0.001
EDD_{HD-MT} (10 ºC days)	-0.062	0.107	0.564
$Prcp_{PT-JT}$ (50 mm)	0.214	0.017	<0.001
$Prcp_{JT-HD}$ (50 mm)	0.189	0.036	<0.001
$Prcp_{HD-MT}$ (50 mm)	0.041	0.022	0.059
$Prcp_{PT-JT}$ Squared (50^2 mm)	-0.014	0.002	<0.001
$Prcp_{JT-HD}$ Squared (50^2 mm)	-0.039	0.007	<0.001
$Prcp_{HD-MT}$ Squared (50^2 mm)	-0.011	0.003	<0.001
HDW_{HD-MT} (10 hours)	-0.091	0.026	<0.001
R-squared	0.590		
p-value (F-test)	<0.001		

Supplementary Table 4. Climate dataset links.

Data sources	Links
ERA5-Land	https://cds.climate.copernicus.eu/cdsapp#!/dataset/reanalysis-era5-land?tab=overview
HadISD	http://www.metoffice.gov.uk/hadobs/hadisd/
Pacific Decadal Oscillation (PDO)	https://psl.noaa.gov/pdo/

Note: PDO data are calculated using the National Oceanic and Atmospheric Administration's Extended Reconstructed SST data set, version 5 (ERSST.v5), on a 2° × 2° latitude-longitude grid.

Supplementary Table 5. Root mean square error (RMSE) for estimated planting (PT) and maturity (MT) dates across South Dakota, Colorado, Oklahoma, and Texas.

South Dakota				Colorado			
Locations		RMSE (days)		Locations		RMSE (days)	
Latitude	Longitude	PT	MT	Latitude	Longitude	PT	MT
43.19°	-99.19°	1	3	37.32°	-102.56°	5	2
43.19°	-100.72°	2	4	37.96°	-103.07°	4	2
43.19°	-101.66°	4	4	37.96°	-102.39°	3	2
43.21°	-98.59°	2	2	38.43°	-102.74°	3	2
43.24°	-103.53°	6	5	38.83°	-102.60°	3	2
43.33°	-97.75°	3	2	38.99°	-103.51°	4	4
43.34°	-102.55°	4	4	39.29°	-104.14°	4	4
43.35°	-99.88°	1	1	39.31°	-102.60°	2	3
43.39°	-98.37°	1	0	39.65°	-104.34°	5	4
43.58°	-100.76°	5	0	39.87°	-104.34°	4	4
43.67°	-98.15°	2	2	39.97°	-103.20°	3	3
43.69°	-101.63°	3	1	40.00°	-102.42°	2	2
43.72°	-98.56°	5	1	40.26°	-103.81°	3	4
43.72°	-99.08°	1	0	40.55°	-104.39°	5	4
43.90°	-99.85°	0	2	40.59°	-102.36°	3	2
43.96°	-100.69°	1	1	40.67°	-105.46°	5	6
44.00°	-102.82°	3	4	40.72°	-103.11°	2	3
44.07°	-98.63°	4	1	40.88°	-102.35°	4	3
44.08°	-99.20°	3	0				
44.29°	-101.54°	0	1				
44.37°	-97.49°	0	2				
44.39°	-100.00°	0	0				
44.41°	-100.74°	0	1				
44.41°	-98.28°	1	1				
44.55°	-99.49°	1	2				
44.55°	-99.00°	1	4				
44.57°	-102.72°	0	2				
44.72°	-100.13°	0	1				
44.86°	-97.73°	2	1				
44.91°	-103.51°	2	4				
44.94°	-98.35°	1	1				
44.98°	-101.67°	2	1				
45.06°	-99.96°	0	0				
45.07°	-99.15°	0	3				
45.16°	-100.87°	3	1				
45.42°	-99.22°	1	2				
45.43°	-100.03°	3	1				
45.49°	-102.48°	2	3				
45.71°	-101.20°	1	4				

Supplementary Table 5. Continued.

Oklahoma				Texas			
Locations		RMSE (days)		Locations		RMSE (days)	
Latitude	Longitude	PT	MT	Latitude	Longitude	PT	MT
33.96°	-96.26°	9	6	28.87°	-99.76°	3	5
34.11°	-97.84°	9	6	28.87°	-99.11°	4	4
34.29°	-98.37°	9	5	29.36°	-99.11°	2	4
34.37°	-98.92°	10	4	29.36°	-99.76°	6	5
34.49°	-97.85°	7	6	29.45°	-98.52°	3	4
34.59°	-99.41°	8	5	29.58°	-97.95°	3	4
34.66°	-98.47°	8	3	30.65°	-97.60°	5	4
34.70°	-97.31°	5	4	30.79°	-96.98°	4	4
34.74°	-99.85°	7	5	31.04°	-97.48°	5	4
34.92°	-98.98°	7	3	31.16°	-98.82°	4	5
34.94°	-99.56°	6	4	31.20°	-99.35°	6	6
35.01°	-97.44°	4	4	31.25°	-96.94°	2	4
35.02°	-97.88°	5	4	31.33°	-99.86°	5	6
35.17°	-98.38°	5	3	31.39°	-97.80°	5	5
35.20°	-97.33°	5	5	31.40°	-100.46°	7	4
35.21°	-96.95°	5	4	31.55°	-97.20°	2	4
35.27°	-99.68°	5	4	31.70°	-98.11°	5	5
35.29°	-98.99°	6	4	31.77°	-99.45°	6	5
35.54°	-97.98°	6	5	31.83°	-99.98°	5	6
35.55°	-97.41°	8	5	31.90°	-97.63°	5	5
35.62°	-95.38°	11	7	31.99°	-97.13°	5	3
35.64°	-99.00°	4	3	32.05°	-96.47°	5	3
35.69°	-99.70°	6	3	32.30°	-99.37°	8	5
35.88°	-98.43°	4	4	32.30°	-99.89°	9	5
35.92°	-97.44°	7	3	32.30°	-100.41°	6	7
35.95°	-97.94°	5	4	32.35°	-96.79°	6	3
35.96°	-95.52°	9	5	32.38°	-97.37°	6	3
35.99°	-99.01°	4	3	32.60°	-96.29°	6	4
36.08°	-96.98°	7	5	32.74°	-99.35°	8	5
36.22°	-99.75°	5	3	32.74°	-99.88°	9	5
36.30°	-95.23°	9	5	32.74°	-102.64°	10	7
36.31°	-98.54°	4	4	32.74°	-100.40°	9	7
36.32°	-96.70°	7	4	32.77°	-96.78°	7	3
36.37°	-95.60°	8	5	33.12°	-96.09°	10	4
36.38°	-97.78°	4	4	33.17°	-102.34°	10	7
36.39°	-97.23°	6	5	33.18°	-98.69°	10	4
36.42°	-99.26°	4	3	33.18°	-99.21°	7	5
36.63°	-96.40°	6	5	33.18°	-99.73°	7	5
36.72°	-95.90°	8	5	33.18°	-100.25°	8	7
36.73°	-98.32°	7	5	33.19°	-96.57°	8	4
36.75°	-101.49°	3	4	33.21°	-97.12°	8	4
36.75°	-102.52°	4	7	33.39°	-95.67°	10	4
36.75°	-100.48°	3	3	33.59°	-96.11°	7	4
36.76°	-95.21°	6	5	33.60°	-102.83°	9	7
36.77°	-98.87°	4	4	33.61°	-99.74°	7	5
36.79°	-99.67°	5	3	33.61°	-101.82°	11	7
36.80°	-97.79°	3	5	33.61°	-101.30°	9	7
36.82°	-97.14°	6	4	33.62°	-98.69°	8	4
36.84°	-94.81°	7	5	33.62°	-99.21°	7	5

Supplementary Table 5. Continued.

Texas				Texas			
Locations		RMSE (days)		Locations		RMSE (days)	
Latitude	Longitude	PT	MT	Latitude	Longitude	PT	MT
33.62°	-100.78°	9	7	34.96°	-100.27°	10	7
33.63°	-96.68°	9	4	34.96°	-101.36°	3	7
33.64°	-97.21°	10	4	34.97°	-101.90°	4	7
33.67°	-95.57°	11	4	34.97°	-102.60°	7	7
33.68°	-97.72°	11	5	35.40°	-100.81°	5	7
33.79°	-98.21°	10	5	35.40°	-100.27°	11	8
33.97°	-99.78°	7	4	35.40°	-101.89°	4	7
33.99°	-98.70°	10	4	35.40°	-101.35°	2	7
34.07°	-102.83°	7	7	35.41°	-102.60°	6	7
34.07°	-102.35°	11	7	35.84°	-100.27°	6	8
34.07°	-101.83°	11	7	35.84°	-101.89°	6	7
34.07°	-101.30°	8	7	35.84°	-100.81°	5	7
34.08°	-100.28°	5	7	35.84°	-101.35°	4	7
34.08°	-99.24°	7	4	35.84°	-102.60°	8	7
34.29°	-99.75°	6	5	36.28°	-101.35°	6	7
34.53°	-100.21°	7	7	36.28°	-101.89°	9	7
34.53°	-102.26°	7	7	36.28°	-100.27°	8	8
34.53°	-102.78°	5	7	36.28°	-102.60°	11	7
34.53°	-101.21°	11	7	36.28°	-100.82°	6	8
34.53°	-101.74°	8	7				

Supplementary References

1. Barber, C. B., Dobkin, D. P. & Huhdanpaa, H. The quickhull algorithm for convex hulls. *ACM Trans. Math. Softw.* **22**, 469-483 (1996).

2. Vehtari, A., Gelman, A. & Gabry, J. Practical Bayesian model evaluation using leave-one-out cross-validation and WAIC. *Stat. Comput.* **27**, 1413-1432 (2017).

3. Robertson, D. L. G., S.O.; Brown, B.D. Southern Idaho Dryland Winter Wheat Production Guide. (University of Idaho: Moscow, ID, USA, 2004).

4. Peairs, F., & Armenta, R. Wheat Production and Pest Management for the Great Plains Region. (Colorado State University Extension, 2010).

5. Lobell, D. B., Sibley, A. & Ortiz-Monasterio, J. I. Extreme heat effects on wheat senescence in India. *Nat. Clim. Change* **2**, 186-189 (2012).

6. Dunn, R. J., Willett, K. M., Parker, D. E. & Mitchell, L. Expanding HadISD: Quality-controlled, sub-daily station data from 1931. *Geosci. Instrum. Methods Data Syst.* **5**, 473-491 (2016).

7. Schneider, T. Analysis of incomplete climate data: Estimation of mean values and covariance matrices and imputation of missing values. *J. Clim.* **14**, 853-871 (2001).

8. Lobell, D. B., Schlenker, W. & Costa-Roberts, J. Climate trends and global crop production since 1980. *Science* **333**, 616-620 (2011).
